# BCAA metabolism in pancreatic cancer affects lipid balance by regulating fatty acid import into mitochondria

**DOI:** 10.1186/s40170-024-00335-5

**Published:** 2024-03-26

**Authors:** Klára Gotvaldová, Jitka Špačková, Jiří Novotný, Kamila Baslarová, Petr Ježek, Lenka Rossmeislová, Jan Gojda, Katarína Smolková

**Affiliations:** 1 Institute of Physiology of the Czech Academy of Sciences, Laboratory of Mitochondrial Physiology, Vídeňská 1083, 142 20, Prague 4 – Krč, Czech Republic; 2 First Faculty of Medicine, Charles University, Prague, Czech Republic; 3 Department of Pathophysiology, Center for Research on Nutrition, Metabolism, and Diabetes, Third Faculty of Medicine, Charles University, Prague, Czech Republic; 4 Third Faculty of Medicine, Franco-Czech Laboratory for Clinical Research on Obesity, Prague, Czech Republic; 5 Department of Internal Medicine, Královské Vinohrady University Hospital and Third Faculty of Medicine, Prague, Czech Republic

**Keywords:** BCAA metabolism, Pancreatic cancer, Mitochondria, Triglycerides, Lipid droplets, Fatty acid/Transport, Fluorescence microscopy, Lipidomics

## Abstract

**Background:**

Pancreatic ductal adenocarcinoma (PDAC) has been associated with the host dysmetabolism of branched-chain amino acids (BCAAs), however, the implications for the role of BCAA metabolism in PDAC development or progression are not clear. The mitochondrial catabolism of valine, leucine, and isoleucine is a multistep process leading to the production of short-chain R-CoA species. They can be subsequently exported from mitochondria as short-chain carnitines (SC-CARs), utilized in anabolic pathways, or released from the cells.

**Methods:**

We examined the specificities of BCAA catabolism and cellular adaptation strategies to BCAA starvation in PDAC cells in vitro. We used metabolomics and lipidomics to quantify major metabolic changes in response to BCAA withdrawal. Using confocal microscopy and flow cytometry we quantified the fluorescence of BODIPY probe and the level of lipid droplets (LDs). We used BODIPY-conjugated palmitate to evaluate transport of fatty acids (FAs) into mitochondria. Also, we have developed a protocol for quantification of SC-CARs, BCAA-derived metabolites.

**Results:**

Using metabolic profiling, we found that BCAA starvation leads to massive triglyceride (TG) synthesis and LD accumulation. This was associated with the suppression of activated FA transport into the mitochondrial matrix. The suppression of FA import into mitochondria was rescued with the inhibitor of the acetyl-CoA carboxylase (ACC) and the activator of AMP kinase (AMPK), which both regulate carnitine palmitoyltransferase 1A (CPT1) activation status.

**Conclusions:**

Our data suggest that BCAA catabolism is required for the import of long chain carnitines (LC-CARs) into mitochondria, whereas the disruption of this link results in the redirection of activated FAs into TG synthesis and its deposition into LDs. We propose that this mechanism protects cells against mitochondrial overload with LC-CARs and it might be part of the universal reaction to amino acid perturbations during cancer growth, regulating FA handling and storage.

**Supplementary Information:**

The online version contains supplementary material available at 10.1186/s40170-024-00335-5.

## Introduction

Pancreatic ductal adenocarcinoma (PDAC) is the most common type of pancreatic tumors. Its incidence and attributable death rate have been increasing continuously over the last decades [1, 2]. One-year mortality of PDAC patients is considerably high and it sparked intensive research on PDAC metabolism to identify metabolic pathways that could be targeted to prevent tumor development. Although enzymes of the branched-chain amino acids (BCAAs) catabolic pathways are generally known, we still lack evidence regarding the catabolic fate of BCAAs in general, or specifically in cancer cells. We currently acknowledge their role in transamination, complete oxidation (and utilization in the TCA cycle), and as metabolic precursors, i.e. BCAA-derived acetyl-CoA can be a precursor of cholesterol, and early BCAA-derived carnitines (BCAA-CARs) can participate in the synthesis of certain types of fatty acids (FAs) [3, 4]. Besides this, BCAAs, mostly leucine, have been implicated in the activation of mammalian target of rapamycin complex 1 (mTORC1) signaling and consequent anabolic program under physiological and pathological conditions [5, 6]. The interference of BCAA catabolism with other metabolic pathways is unexplored and the consequences of such crosstalk, especially at the level of short-chain carnitines (SC-CARs), which originate from the respective R-CoAs [7] remain completely unknown.

Several works have focused also on the role of BCAA catabolism in PDAC growth, but it is not entirely clear how BCAA catabolism contributes to cancer development and to what extent. Using a KC and KPC genetic model, Carrer et al. 2019 [8] showed that BCAAs are metabolized into acetyl-CoA, especially in the early stages of tumor development, i.e. PanIN lesions. Li et al. [9] demonstrated the role of mitochondrial branched-chain amino acid transaminase 2 (BCAT2) in PDAC development and showed that its genetic deletion decreased transamination activity and respiration [9], similarly to dihydrolipoamide branched chain transacylase E2 (DBT) knockdown [10]. It was also demonstrated that BCAT2 in cancer cells reaminates branched-chain keto acids (BCKAs) supplied by cancer-associated fibroblasts into BCAAs. Lei et al. [11] showed that acetylation promotes BCAT2 degradation to suppress BCAA catabolism and pancreatic cancer growth. On the other hand, the overexpression of BCAT2 renders cells and xenografted tumors insensitive to ferroptosis induction by improving glutathione homeostasis [12]. In summary, several settings of BCAA utilization in PDAC have been described, but it is possible that the in situ situation adapts according to the actual nutrient and desmoplastic circumstances, and the whole-body substrate distribution.

It is pertinent to presume that disturbances in the BCAAs supply or intake affect cellular behavior and possibly also cancer biology. For instance, mouse chow low in BCAAs delayed the development of PanIN in KC mice and PDAC progression in KPC mice [9]. Moreover, diet containing high levels of BCAA promoted PanIN in KC mice owing to BCAT2 stabilization via USP1 deubiquitinylase [13]. At the moment, we cannot conclude whether any potential BCAA utilization in cancer cells is a matter of the essential nature of BCAAs in protein synthesis, or whether the effect is also metabolic. To resolve this issue, we performed an in-depth analysis of BCAA metabolism in PDAC cultured cells. We report here that a lack of BCAAs can trigger adaptive responses similar to cellular starvation programs, leading to lipid droplet (LD) accumulation. Thus, we describe a novel mechanism of FA redirection towards neutral lipid synthesis, which also affects mitochondrial bioenergetics. In our model, BCAAs are involved in the regulation of mitochondrial capacity to oxidize FAs by β-oxidation, probably via carnitine palmitoyltransferase 1A (CPT1). Moreover, our work implies the role of SC-CARs derived from BCAAs as mediators of downstream metabolic remodeling after nutrient exhaustion.

## Materials and methods

### Cell cultures

In this study, we used commonly used human cultured cancer cell lines PaTu 8902, MIA PaCa-2, and PANC-1, the authenticity of which was confirmed by STR profiling (Baria, Czech Republic). Cells were grown in 1×RPMI *w/o* phenol red containing 10% FBS (Biosera), 1.5 g·L^−1^ of NaHCO_3_, 10 mM HEPES, 1×penicillin/streptomycin, 1 mM sodium pyruvate, 5 mM glucose, 1 mM glutamine, and optionally 200 µM of valine, leucine, and isoleucine (each). Treatment under -BCAA conditions was for 48–72 h according to the requirements of the experiment, i.e. 72 h for cell viability testing and 48 h for other experiments. As a rule, the medium was replaced with a fresh one a day before the experiment.

### Analytical methods

For the analysis of BCAA-derived carnitine species, we optimized the LC-MS based method in collaboration with the metabolomics facility at the Laboratory of Mass Spectrometry at the BIOCEV Research Center; Faculty of Science, Charles University. 10^6^ cells were used as a starting point. Cell pellets were washed with 137 mM NaCl and 2.7 mM KCl, and subsequently resuspended in a buffer containing 40% acetonitrile, 40% methanol, and 0.1% (*v/v*) formic acid in distilled water. Samples were incubated and shaken for 20 min on ice, centrifuged for 15 min at 16,000×g, and the supernatants were evaporated and resuspended in 40 µl of 10% acetonitrile containing 1 µg/ml indole-2,4,5,6,7-d_5_-3-acetic acid (CDN Isotopes) as the internal standard. The sample volume for injection was 5 µl. Samples were analyzed in a Dionex Ultimate 3000RS liquid chromatography system coupled to a TSQ Quantiva mass spectrometer (ThermoScientific) using an ESI source in positive mode with the following ion source parameters: ion transfer tube temperature 325 °C, vaporizer temperature 350 °C, spray voltage 3500 V, sheath gas 50, and aux gas 10. A Luna Omega PS C18 (150 × 2.1 mm, 3 μm, 100 Å) (Phenomenex, Torrance, CA, USA) was used for the separation of analytes. The column was maintained at 35 °C. The buffer composition was A: 10 mM ammonium formate in water, 0.1% F.A, 0.01% heptafluorobutyric acid; B: 99.8% methanol, and the flow rate was 0.3 ml/min and total run time was 24 min. The elution gradient (A/B) started at 0–2 min: 100% A; 3–9 min: 85% A; 9.5–14 min: 65% A; followed by a washing phase 14.1–17 min: 0% A, and equilibration phase 17.1–24 min: 100% A. Selective reaction monitoring (SRM) was used for the detection and quantification of selected compounds. Data were processed in the Skyline software.

For the targeted metabolomics of TCA and mitochondrial metabolites, we used previously reported GC-MS based methods. Cells were rinsed with PBS, trypsinized, counted, snap-frozen, and stored at -80 °C until analysis. At least 10^6^ cells per sample were used. Cell metabolites 2OG (347.3), fumarate (245.2), malate (335.3), and citrate (273.2) were measured using a method reported previously [14, 15]. In addition, the analytes succinate (247.2), aspartate (232.2), alanine (190.1), and glutamate (246.2) were considered in this method based on the molecular ions (in brackets) found in the NIST mass spectral library specific for EI ionization. DL-malic acid-2,3,3-d_3_ (338.3, 10 µl of 100 µg/ml) was used as the internal standard. The total analysis time was 10 min.

For lipidomics and metabolomics, cells were grown on 6-well plates and treated as required, quickly washed with PBS, snap-frozen, and stored at -80 °C. Metabolites were extracted using a biphasic solvent system of cold methanol, methyl *tert*-butyl ether, and water [16]. An aliquot of the bottom (polar) phase was collected, cleaned up using an acetonitrile/isopropanol mixture, and after evaporation, the dry extract was resuspended in 5% methanol with 0.2% formic acid followed by separation in an Acquity UPLC HSS T3 column (Waters). Another aliquot of the bottom phase was evaporated, resuspended in an acetonitrile/water mixture, and separated in an Acquity UPLC BEH Amide column. Metabolites were detected in negative and positive electrospray ion mode (Thermo Q Exactive Plus instrumentation) [17]. Signal intensities were normalized to the respective total ion count (TIC) before subsequent statistical analysis. TIC-normalized intensities are provided in Supplementary Material 2.

### Flow cytometry

Cells were seeded on 12-well plates 72 h prior to the experiment. BCKA and CHS-FBS were added 48 h before analysis, while other treatments (DGAT1i, DGAT2i, 3-MA) were added *o/n*, or for 3 h prior analysis (BafA, CQ). Cells were stained with 500 nM BODIPY™ 493/503 (ThermoFisher Scientific) in PBS for 10 min, washed with PBS, and harvested by trypsinization. Cell pellets were resuspended in PBS containing 0.5 mM EDTA, stained with HOECHST 33258 (18 µM, MERCK), and fluorescence intensity was measured using a BD LSRII flow cytometer with excitation at 488 nm and emission at 500–550 nm. The FITC-A fluorescence intensities of BODIPY™ 493/503 of each live cell (HOECHST 33258-negative) were analyzed using the FlowJo software (LLC) and expressed as median fluorescence intensity, which were integrated into the final graphs.

### Real-time PCR

RNA was isolated from fresh cells using a Total RNA Purification Kit (Jena Bioscience), followed by reverse transcription using a TATAA GrandScript cDNA Synthesis Kit (TATAA Biocenter). Q-PCR was performed using Forget-Me-Not EvaGreen qPCR Master Mix (Biotium) and a CFX Connect Real-Time PCR System (BioRad). Calculated crossing points were used to calculate ∆∆Ct values (compared to control cells). Primer sequences are summarized in Table S1 in Supplementary Material 1.

### Western blotting

Cells were lysed in a cell lysis buffer (50 mM Tris-HCl, pH 7.5, 2 mM EDTA, 150 mM NaCl, 1% Triton X-100) containing 1mM phenylmethylsulfonyl fluoride (PMSF), 1% protease inhibitor cocktail for 30 min on ice. The lysates were then centrifuged at 6,000×g for 5 min at 4 °C. 10–50 µg of protein was separated through 8–12% SDS-PAGE gel. SDS-PAGE electrophoresis was carried out at 75 V for 30 min, followed by 150 V for 60 min. Proteins separated in SDS-PAGE gels were transferred to polyvinylidene difluoride (PVDF) membranes using a tank transfer system conducted at 300 mA for 75 min. Subsequently, following the manufacturer’s recommendations for specific antibodies, PVDF membranes were blocked, rinsed, and incubated with primary and secondary antibodies. Finally, proteins were detected using chemiluminescence method with the ChemiDoc MP Imaging System (Bio-Rad).

### Viability

Cells were plated on 96-well plates to a density of 5,000–10,000 cells per well. 24-hours post-seeding, medium was changed and inhibitors added. After another 72 h, neutral-red assay was performed [18].

### Respirometry

High-resolution respirometry (HRR) was applied with intact cells using a coupling control protocol (CCP) and O2k-respirometer (Oroboros Instruments, Austria), as reported previously [19]. Cells were measured in the respective growth medium. For the β-oxidation of intact cells, the CCP protocol was used, but cells were resuspended in a growth medium containing 1% FBS.

### Preparation of BSA-palmitate conjugate

Palmitate conjugated with BSA was prepared as follows. First, a 0.17 mM solution of Albumin Bovine Fraction V, Protease and Fatty Acid-Free (Serva, Serving Scientists) was prepared in 150 mM NaCl and stirred until BSA dissolved at 37 °C. Then 1 mM sodium palmitate was prepared into 150 mM NaCl solution by stirring at 70 °C. The solutions were gradually combined with stirring, incubated for 1 h at 37 °C in a bath, then buffered to pH 7.4 and made up to a final volume of 1 mM palmitate: 0.17 mM BSA. For respiration measurements, this 5×solution was diluted with 2×RPMI medium to a final ratio of 200 µM palmitate: 34 µM BSA and cells were incubated *o/n*.

### Confocal microscopy

Cells were cultured for 3 days to 70–80% cell confluency on quartz coverslips coated with poly-l-lysine. The cells were then stained with 500 nM BODIPY™ 493/503 (ThermoFisher Scientific) along with 1 µM HOECHST 33258 for 15 min at 37 °C. Alternatively, for mitochondrial import experiments, cells were stained with 500 nM BODIPY™ FL C_16_ (ThermoFisher Scientific) in the cultivation medium *o/n* and subsequently stained with 500 nM MitotrackerRed™ for 15 min at 37 °C. The coverslips were washed twice in a PBS buffer and then placed in a thermostable chamber supplied with 5% CO_2_ and measured by confocal microscopy (Leica TSC SP8 AOBS WLL MP) in the regular cultivation medium. An HC PL APO 63×/1.20 NA W CORR CS2, WD = 0.3 mm objective was used for observation with argon laser excitation (488 nm) for both BODIPY dyes (BODIPY™ 493/503, and BODIPY™ FL C_16_) with a 500–550 nm emission filter. For MitotrackerRed (581⁄644), we used a WLL laser with excitation at 581 nm and emission of 620–660 nm. An argon laser with excitation at 405 nm and emission filter of 415–480 nm was used for HOECHST 33258. The results were evaluated using the Fiji ImageJ software as mean of BODIPY 493/503 fluorescence related to HOECHST 33342 (%).

### Statistical analysis

All data represented as bar plots are expressed as means and standard deviations (SD). Whisker plots are depicted from minimum to maximum values. Statistical significance was calculated using GraphPad Prism version 9.3.1 for Windows, (GraphPad Software, San Diego, California USA, www.graphpad.com) using one-way or two-way analysis of variance (ANOVA) followed by multiple comparison testing (Tukey’s, Dunnett’s, and Sidak’s, recommended by software). N stands for number of independent experiments, n stands for sample size. Statistically significant *p*-values are presented in figures as *p < 0.05, **p < 0.01, ***p < 0.001, ****p < 0.0001. For volcano plots, *p*-values were calculated using Welch’s test in RStudio, version 1.4, using the matrixTests package https://CRAN.R-project.org/package=matrixTests. Multivariate analyses were performed in Workflow4metabolomics [20] or MetaboAnalyst 5.0 0 [21], as specified in the respective figure legends.

### Chemicals

We used the following specific chemical compounds for cell treatment.

#### Chemicals and standards

- O-Acetyl-L-carnitine hydrochloride MERCK A6706
- Propionyl-L-carnitine (Acar 3:0) MERCK 91275
- Tigloyl-L-carnitine (Acar 5:1) MERCK 39588
- Isobutyrylcarnitine (Acar 4:0) MERCK 91299
- 3-Methylcrotonyl-L-carnitine (Acar 5:1) MERCK 28986
- Isovaleryl-L-carnitine (Acar 5:0) MERCK 91403
- 2-Methylbutyryl-L-carnitine (Acar 5:0) MERCK 91519
- Butyryl-L-carnitine (Acar 4:0) MERCK 08984
- Valeryl-L-carnitine (Acar 5:0) MERCK 04265
- (±)-Sodium β-hydroxyisobutyrate MERCK 36105
- (±)-3-Methyl-2-oxovaleric acid sodium salt (KMV) MERCK K7125
- Sodium 3-methyl-2-oxobutyrate (KIV) MERCK 198994
- Sodium 4-methyl-2-oxovalerate (KIC) MERCK K0629
- Phenylmethylsulfonyl fluoride (PMSF) MERCK 329-98-6
- Protease inhibitor cocktail MERCK P8340

#### Treatments

- ETO, carnitine palmitoyltransferase 1A inhibitor, MERCK 236020, final concentration 50 µM
- DGAT1i, diacylglycerol-O-acetyl-transferase 1 inhibitor A922500, MERCK A1737, final concentration 10 µM
- DGAT2i, diacylclycerol-O-acetyl-transferase 2 inhibitors PF-06424439, MERCK PZ0233, final concentration 20 µM
- 3-MA, autophagosome inhibitor 3-Methyladenin, MERCK M9281, final concentration 1 mM
- BafA, autolysosome formation inhibitor Bafilomycin A1, Abcam ab120497, final concentration 100 nM
- CQ, autolysosome formation inhibitor chloroquine, MERCK C6628, final concentration 100 µM
- ACCi, 5-(tetradecyloxy)-2-furoic acid (TOFA), Abcam ab141578, final concentration 10 µM
- AICAR, 5-Aminoimidazole-4-carboxamide 1-β-D-ribofuranoside, MERCK A9978, final concentration 500 µM
- AMPKi, Compound C, MERCK 171260, final concentration 10 µM
- L-carnitine hydrochloride, MERCK C0283, final concentration 1 mM
- BCKA, i.e. KIC (MERCK K0629), KIV (MERCK 198994), KMV (MERCK K7125), final concentration 200 µM

### Antibodies

- anti BCAT2 rabbit pAb, AB Clonal A7426
- anti BCKDHA rabbit pAb, ABL Clonal 49806
- anti-PDHA1 mouse mAb, Invitrogen 459400
- anti-PDHA1 (phospho S293) rabbit pAb, Abcam 92696
- anti-ATP5A mouse mAb, Abcam ab14748
- anti-Tim23 mouse mAb, Abcam ab176558
- anti-Gls1 rabbit mAB, Abcam ab156876
- anti-AMPKα rabbit pAb, Cell Signaling #2532
- anti-AMPKα (phospho Thr172) rabbit pAb, Cell Signaling #2531
- anti-Rabbit IgG–Peroxidase antibody produced in goat, MERCK A0545
- anti-Mouse IgG–Peroxidase antibody produced in goat, MERCK A4416

### Fluorescent dyes

- BODIPY™ 493/503 (4,4-Difluoro-1,3,5,7,8-Pentamethyl-4-Bora-3a,4a-Diaza-s-Indacene) ThermoFisher Scientific D3922
- BODIPY™ FL C_16_ (4,4-Difluoro-5,7-Dimethyl-4-Bora-3a,4a-Diaza-s-Indacene-3-Hexadecanoic Acid), ThermoFisher Scientific D3821
- MitoTracker™ Red FM, 581/644, ThermoFisher Scientific M22425
- Hoechst 33342, ThermoFisher Scientific 62249

## Results

### Oxidation of BCAAs in cultured PDAC cells

**Fig. 1:**
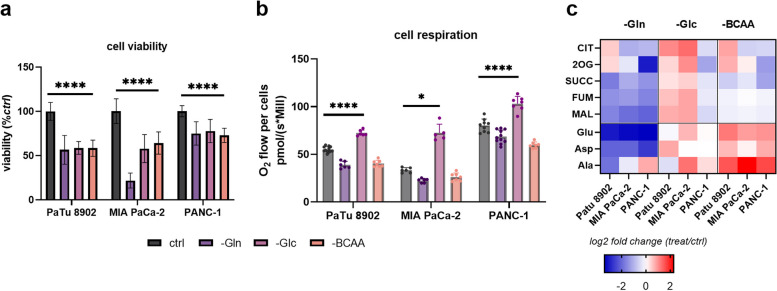
BCAA starvation affects cell growth and mitochondrial metabolism. **a** Cell viability assay comparing no glucose (-Glc), no glutamine (-Gln), and no BCAA (-BCAA) conditions after 72 h of starvation. Two-way ANOVA followed by Tukey’s multiple comparisons tests. *N* > 4, *n* > 50. Criteria of significance: *p < 0.05; **p < 0.01; ***p < 0.001, ****p < 0.0001. **b** Respiration of intact cells measured by high-resolution respirometry using indicated cell lines and starvation conditions, including BCAA. Cells were starved of BCAAs for 48 h prior experiment. Two-way ANOVA followed by Tukey’s multiple comparisons tests. *N* > 3, *n* ≥ 6. Criteria of significance: *p < 0.05; **p < 0.01; ***p < 0.001, ****p < 0.0001. **c** Levels of mitochondrial metabolites measured by GC-MS expressed as a log2 fold change compared to complete medium (*ctrl*). Cells were starved 48 h prior the experiment. Citrate (CIT), 2-oxoglutarate (2OG), succinate (SUCC), fumarate (FUM), malate (MAL), glutamate (Glu), aspartate (Asp), and alanine (Ala)

To estimate the overall contribution of valine, leucine, and isoleucine to the viability of PDAC-derived cultured cells (PaTu 8902, MIA PaCa-2, and PANC-1), we cultivated cells in a BCAA-depleted medium for 72 h and measured their viability in comparison to glucose-free and glutamine-free conditions. Similarly to glucose and glutamine starvation, BCAA depletion (-BCAA) inhibits the cell growth and cell survival of all three tested cell lines (Fig. 1a), but the effect was not additive with glutamine or glucose removal (not shown). To address the question of whether BCAAs are oxidative substrates that contribute to TCA cycle turnover, we measured the respiration parameters of three cancer cell lines under BCAA-depleted conditions. BCAA starvation led to a decrease in respiratory rate, basal (Fig. 1b) and maximum (Fig. S1a), in all three PDAC-derived cell lines, except for MIA PaCa-2 at maximal respiration. Elevated respiration and TCA cycle intermediates in glucose-starving cells can be attributed to partial derepression of Warburg and Crabtree effects [22]. The BCAA depletion resulted in a slight decrease in TCA intermediates, but not in the level of free amino acids involved in mitochondrial transamination reactions, i.e. alanine, glutamate, and aspartate (Fig. 1c). Neither mitochondrial network shape (Fig. S1c), nor expression of mitochondrial proteins specific for distinct sub-compartments (BCKDHA, BCAT2, TIM23, ATP5A, PDH, P-PDH, GLS1; Fig. S1d) was affected under BCAA starvation, but we were able to detect some downregulation in the expression of BCAA catabolizing genes (Fig. S1b). At this point, we cannot conclude whether BCAAs are completely oxidized in PDAC cultured cells in vitro or whether they support other pathways oxidizing substrates in mitochondria, causing respiration decrease. Because we have verified that PDAC cells in BCAA-depleted conditions are viable and consume oxygen by respiration, we consider those conditions as a valid model for the subsequent experiments.

### BCAA-free conditions induce metabolic changes in mitochondrial and extra-mitochondrial pathways

**Fig. 2:**
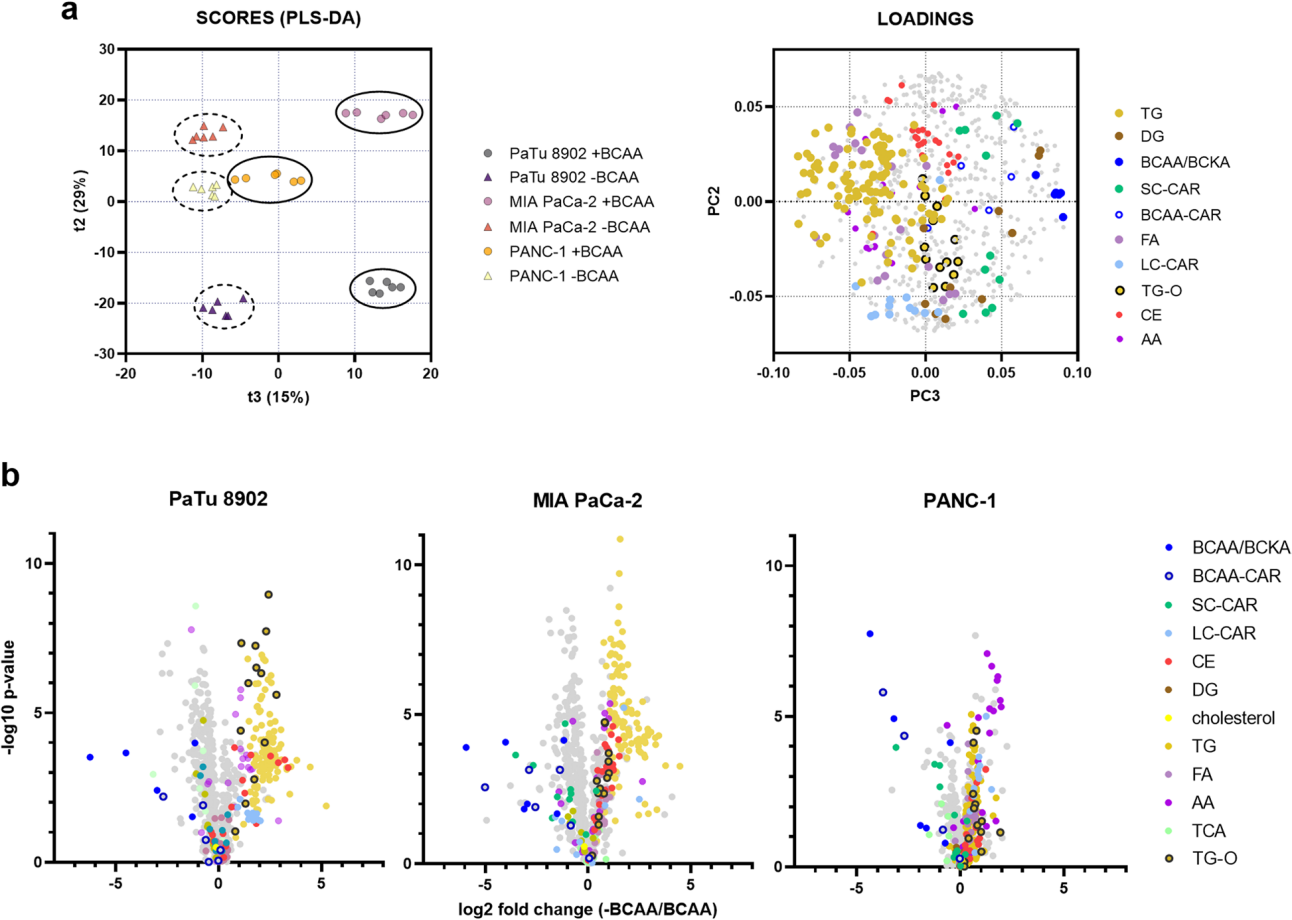
BCAA starvation induces metabolic remodeling. **a** Partial least squares discriminant analysis (PLS-DA) scores plot (left) depicting the stratification of samples based on the presence/absence of BCAAs in PC3 (t3). PLS-DA was performed in Workflow4metabolomics using TIC-normalized data. The loadings plot corresponding to PLS-DA (right) shows a negative correlation between BCAA/BCKA and TG species. **b** Volcano plots of -BCAA-treated cells compared to +BCAA treatment depicting several lipid and metabolic classes, i.e. BCAA/BCKA, short-chain carnitines (SC-CAR), BCAA-derived carnitines (BCAA-CAR), long-chain carnitines (LC-CAR), cholesteryl-esters (CE), diacylglycerols (DG), cholesterol, triglycerides (TG), ether-linked TG (TG-O), free fatty acids (FA), free amino acids (AA), and TCA-cycle metabolites (TCA). Cells were starved 48 h prior the experiment. *P*-values were calculated by Welch’s t-test using the matrixTests package in RStudio

Aside from a role in oxidation or transamination, BCAA catabolism is implicated in the synthesis of odd-chain and branched-chain FAs in adipose tissue [3, 4], FAs, triglycerides (TGs) [23], and cholesterol [8] in PDAC. We asked whether these metabolic pathways are affected by the absence of BCAAs in PDAC. To analyze the metabolic remodeling related to BCAA depletion in a complex way, we performed metabolomics and lipidomics analyses of extracts from cells treated under BCAA-depleted conditions and searched for common features between the cell lines. First, multivariate analyses of polar and non-polar analytes resulted in the stratification of -BCAA and +BCAA groups in the principal component 3 (PC3, t3) (Fig. 2a). The metabolites related to PC3 are presented in the loadings plot and include analytes of metabolic pathways TGs, ether-linked TGs (TGs-O), cholesteryl-esters (CEs), and free amino acids, while among the downregulated metabolites there were downstream metabolites of BCAA catabolism, including SC-CARs and BCAA-CARs. Changes in those pathways were also identified by univariate analyses and highlighted in volcano plots (Fig. 2b). SC-CARs (including BCAA-CARs) are synthesized from their respective R-CoA intermediates produced in the BCAA catabolic pathway by mitochondrial carnitine O-acetyltransferase (CRAT, EC 2.3.1.7) and represent precursors for anabolic functions of BCAAs as previously demonstrated [3, 4]. Lipids most significantly and consistently affected by BCAA starvation were TGs (*p* < 0.001, 5.24⨉, 3.22⨉, and 1.54⨉ for PaTu 8902, MIA PaCa-2, and PANC-1, respectively; Fig. S2).

### The formation of lipid droplets and autophagy is upregulated under BCAA-depleted conditions

**Fig. 3:**
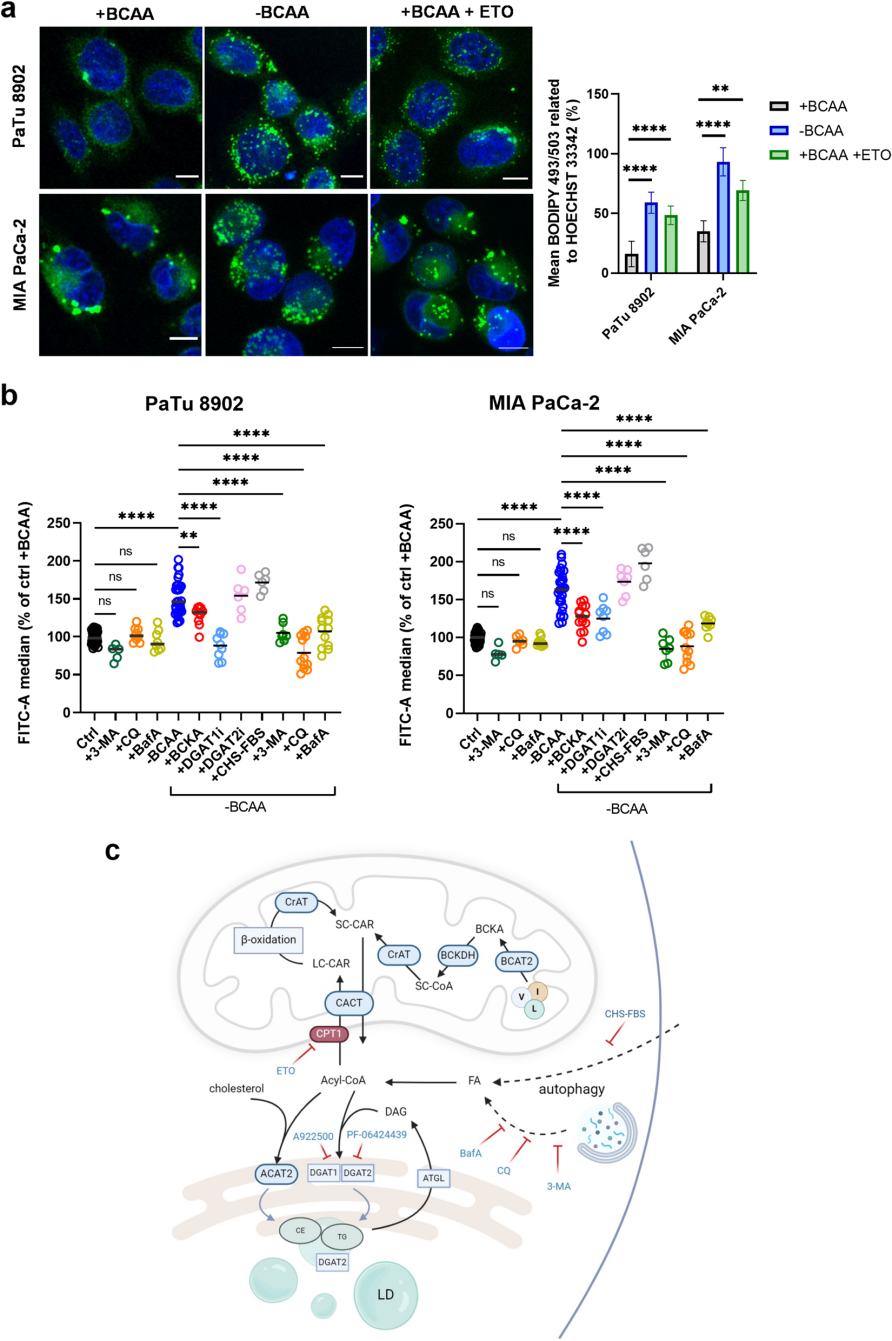
Triglyceride synthesis and lipid droplets are upregulated in PDAC cells starved of BCAAs. **a** Left, imaging of neutral lipids using BODIPY™ 493/503 (green) and nuclei using Hoechst 33342 (blue) depicts build-up of lipid droplets in PaTu 8902 and MIA PaCa-2 cells. Right, the quantification of BODIPY™ 493/503 fluorescence intensity expressed as as per-cent of HOECHST 33342. *N* = 2. *n* ≥ 4. One-way analysis of variance (ANOVA) followed by Tukey’s multiple comparisons tests. **b** Flow cytometry of PaTu 8902 (up) and MIA PaCa-2 (down); BODIPY™ 493/503 mean fluorescence intensities calculated from 10^4^ events. Cells were starved of BCAA for 48 h prior experiment. Color coding: black for control, green for 3-MA, orange for CQ, corn yellow for BafA, blue for -BCAA, red for BCKA, light blue for DGAT1i, pink for DGAT2i, grey for charcoal-stripped serum. One-way ANOVA followed by Tukey’s multiple comparisons tests. *N* > 3, *n* ≥ 6. Criteria of significance: *p < 0.05; **p < 0.01; ***p < 0.001, ****p < 0.0001. **c** Working scheme (right) depicting inhibitors used in the experiments, potentially causative in LD formation. Carnitine palmitoyltransferase 1A inhibitor (CPT1, etomoxir (ETO), 50 µM), diacylglycerol-O-acetyl-transferase 1 inhibitor (DGAT1i, A922500, 10 µM), diacylglycerol-O-acetyl-transferase 2 inhibitor (DGAT2i, PF-06424439, 20 µM), autophagy initiation inhibitor 3-methyladenine (3-MA, 1 mM), autophagy inhibitor bafilomycin A1 (BafA, 100 nM), autophagy inhibitor chloroquine (CQ, 100 µM), charcoal-stripped FBS (CHS-FBS). Created with BioRender.com

We further attempted to explain mechanisms that lead to a build-up of TGs in response to BCAA depletion. To confirm and extend lipidomics data, we used the staining of TGs with the fluorescent probe BODIPY™ 493/503 (BODIPY further on) using flow cytometry and confocal microscopy. Imaging experiments showed a build-up of LDs in -BCAA cells, especially in MIA PaCa-2 and PaTu 8902 (Fig. 3a, b, S3b). Interestingly, the carnitine palmitoyltransferase 1A (CPT1) inhibitor etomoxir (ETO) also caused LD accumulation, suggesting that we were observing the redirection of the FA pool away from mitochondria and toward TG synthesis. Indeed, the inhibition of diacylglycerol O-acyltransferase 1 (DGAT1), one of the enzymes catalyzing the final step of TG synthesis, led to downregulated LD formation in MIA PaCa-2 and PaTu 8902 (Fig. 3b, c, S4e), as quantified by flow cytometry (Fig. S3a). DGAT2 inhibition was ineffective to block LD formation, though. Inhibition of FA import into the cells by treatment with charcoal-stripped serum (CHS-FBS) for 48 h did not lead to a significant decrease in LD formation compared to the -BCAA treatment, suggesting that TGs are synthesized from endogenous FAs. Hence, we inhibited autophagosome formation by 3-methyladenine (3-MA) and autolysosome formation by bafilomycin A (BafA) and chloroquine (CQ) in order to decrease the availability of FAs originating from degraded membranes [24]. 3-MA, BafA, and CQ effectively decreased BODIPY fluorescence in -BCAA but not in +BCAA samples, indicating that autophagy occurs during BCAA starvation, and contributes to LD build-up by releasing FAs. BCAA starvation, therefore, resembles the other previously reported model of nutrient starvation (Hanks balanced salt solution, HBSS) [25–27]. Almost no changes in gene expression of enzymes catalyzing LD formation were observed in BCAA-depleted PDAC cells (Supplementary Fig. S3c). We conclude that BCAA depletion induces TG synthesis via a DGAT1-dependent pathway, and that autophagy is initiated, as demonstrated using treatment with autophagy inhibitors. Importantly, BCKA supplementation stalled LD formation under BCAA-free conditions, which suggests that BCAA-derived metabolites and BCAA catabolism, but not the BCAAs itself, imply the effect of TG accumulation (Fig. 3b, S3b).

### Import of FAs into mitochondria from the cytosol is inhibited in BCAA-free conditions

**Fig. 4:**
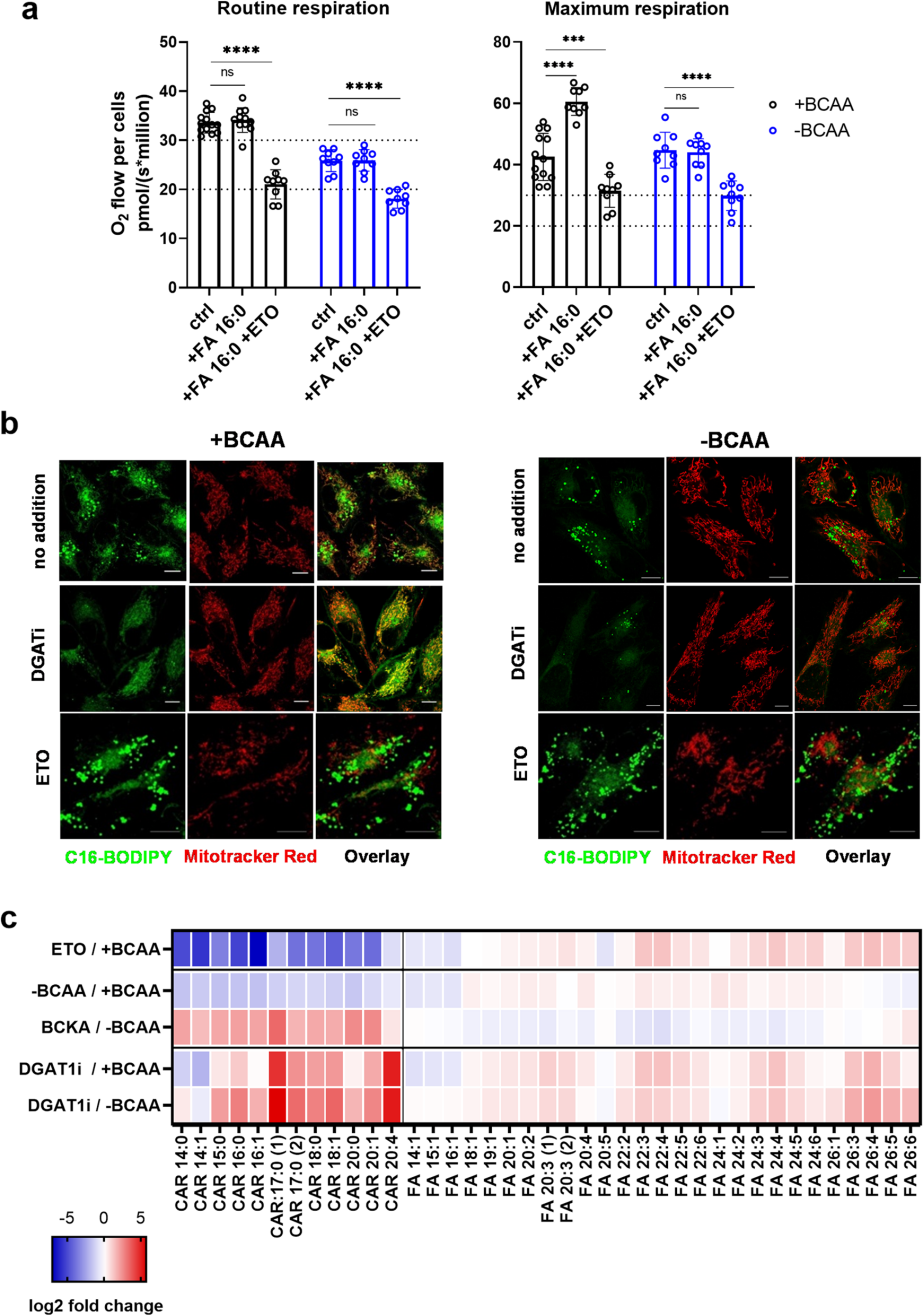
FA import into mitochondria is diverted into TG synthesis in BCAA-starved cells. **a** Routine or maximum respiration of intact cells (PaTu 8902) measured using high-resolution respirometry. Respiratory rates of BSA-palmitate (FA 16:0) with or without ETO addition demonstrate the difference between -BCAA and +BCAA treatment. The assay was performed in RPMI containing 1% FBS and BSA-palmitate (34 µM BSA, 200 µM palmitate). One-way ANOVA followed by Tukey’s multiple comparisons tests. *N* > 3, *n* ≥ 9. Criteria of significance: ***p < 0.001, ****p < 0.0001. **b** Confocal microscopy imaging using fluorescent FA conjugate (BODIPY FL C_16_, C16-BODIPY, green) and a mitochondrial network (Mitotracker™ Red) shows the import of fluorescently-labeled palmitate into mitochondria only with BCAA-containing treatment, which was augmented in DGAT1i-treated cells (inhibition of TG synthesis). No import of C16-BODIPY was observed under BCAA-depleted conditions. ETO treatment inhibits the import of C16-BODIPY into mitochondria and induces LD formation. **c** Log2 fold change of LC-CAR and FA species under designated conditions, namely -BCAA, +BCAA, ETO treatment, and DGAT1i treatment

Previous works reported the delivery of lipolysis-derived FA import into mitochondria in starvation models [26, 28, 29], so we asked whether BCAA levels interfere with the dynamics of FA activation and its subsequent storage vs. oxidation. To determine the functional consequences of BCAA-FA interplay, we used high-resolution respirometry in order to estimate β-oxidation stimulated by BSA-palmitate in the presence and absence of BCAAs. The inability to deliver of LC-CARs into the matrix space would result in the lower respiratory rate. The shortcoming of this approach is that the respiration stimulated by BSA-palmitate is not usually recognized in the routine state, but clear differences can be expected in the maximum respiratory rates (see scheme related to “Palmitate oxidation stress test kit” by Agilent Technologies). Indeed, maximum respiration after the addition of FA 16:0 was lower in BCAA-starved cells, and ETO treatment was able to inhibit respiration to a larger extent in BCAA-containing medium (Fig. 4a, S4a). This experiment suggests that LC-CARs reach the mitochondrial matrix at a lower rate in BCAA-starved cells, and that β-oxidation is functionally intact in control cells.

To further elaborate on our hypothesis, we used fluorescent FA conjugate (BODIPY FL C_16_, C16-BODIPY from here on) and traced its cellular fate. As expected, treatment with ETO led to the deposition of FA conjugate into LDs (Fig. 4b). Vice versa, treatment with DGAT1i induced the import of fluorescent palmitate into mitochondria. However, the import into mitochondria was considerably mitigated in BCAA-depleted conditions, and it was rescued by BCKA or BCAA supplementation (as elaborated in the next chapter). Both imaging and bioenergetics data consistently show the defect in mitochondrial delivery of FA intermediates in BCAA-depleted conditions. A reduced C16-BODIPY staining of the mitochondrial reticulum suggests the inhibition of the CPT1 and/or carnitine-acylcarnitine transferase (CACT) but not inhibited β-oxidation or carnitine palmitoyl transferase 2 (CPT2).

To support the conclusions of imaging data, we performed a lipidomics profiling of MIA PaCa-2 cells cultivated in +BCAA and -BCAA conditions treated with DGATi and ETO. We expected that the suppression of CPT1 function would result in LC-CAR decline, while suppression of β-oxidation or CACT [30] would result in accumulated LC-CAR (Fig. S4b). First, treatment with DGAT1i induced a considerable amount of LC-CARs and FAs (Fig. 4c), although only FA levels were higher in BCAA-starved relative to BCAA-supplied cells treated with DGAT1i (Fig. S4d). As proof-of-concept, we also treated cells with ETO and observed a massive downregulation in LC-CARs along with upregulation of FAs and TGs (Fig. 4c, S4e). Likewise, BCAA depletion also resulted in low LC-CAR levels and high FA levels, which was effectively rescued by BCKA addition. It is also worth noting that CPT1 inhibition leads to the accumulation of TGs with higher molecular weight (54 carbons and more, Fig. S4e), while BCAA shortage, interestingly, led to a uniform change in the number of carbons and the level of saturation.

Based on the imaging and bioenergetics evaluations, we conclude that BCAA starvation compromises the transport of LC-CARs into the mitochondrial matrix via CPT1 and/or CACT inhibition, hence the pool of activated FA (R-CoA) would be redirected into TG synthesis. However, considering the LC-CAR decline and the diminished mitochondrial targeting of C16-BODIPY in BCAA starvation, we favor the possibility that CPT1 rather than CACT inhibition contributes to LD build-up.

### BCAA-derived short-chain acylcarnitines link mitochondrial BCAA metabolism and TG metabolism

**Fig. 5:**
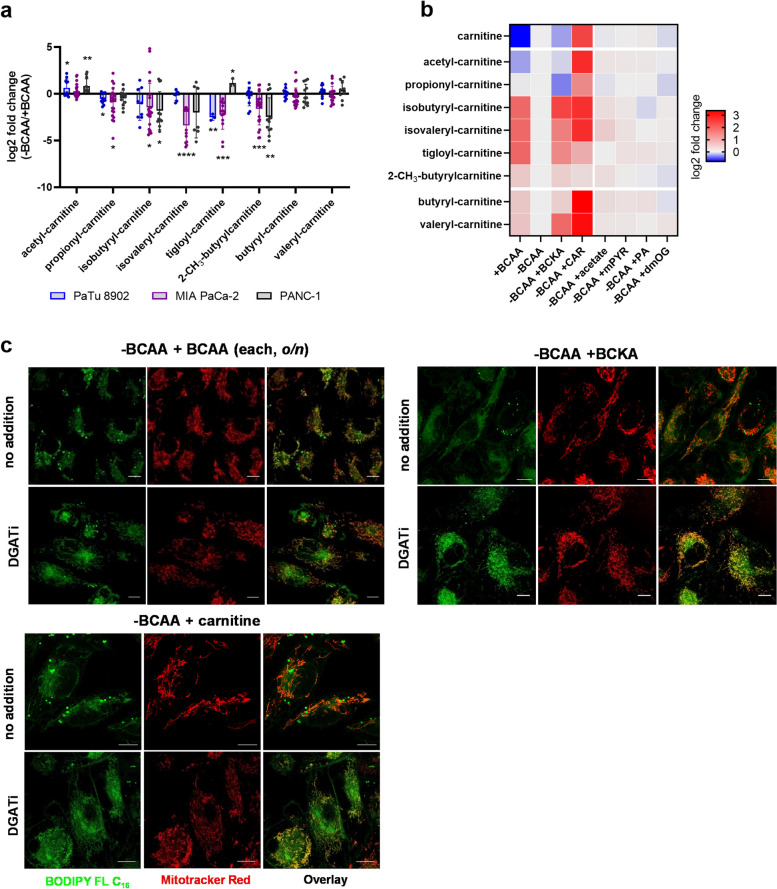
BCAA-derived carnitines respond as regulators of LC-CAR oxidation. **a** Log2 fold change in BCAA-CARs with -BCAA treatment quantified by LC-MS method. Paired t-test. *N* > 3, *n* ≥ 9. Criteria of significance: ***p < 0.001, ****p < 0.0001. **b** Rescue effect of BCAA-CARs under BCAA-depleted conditions with BCKA, free carnitine, and other compounds relevant to mitochondrial metabolism. **c** Confocal microscopy demonstrating the rescue effect of mitochondrial FA import with BCAA (*o/n*), BCKA (48 h), and free L-carnitine (1 mM, *o/n*) treatment

Next, we intended to confirm the shortage of BCAA-CARs under -BCAA conditions. Alternatively, there could be other metabolites that are decreased under the -BCAA conditions and which regulate LC-CAR import into mitochondria. In the PLS-DA analysis (Fig. 2a, b), the stratification of TG and BCAA/BCKA species in the PC3 extremities implies that there is a negative correlation between BCAA and TG metabolism. Therefore, we performed a correlation analysis of TGs with all individual metabolites/lipids available in our data set, calculated Pearson coefficients, and searched for negative correlations, because the supplementation of such metabolites should theoretically rescue the import of C16-BODIPY under the BCAA-depleted conditions. To analyze the rescue effect of the tested compounds on SC-CAR levels, we optimized the LC-MS-based method for the detection of BCAA-CARs, namely isovaleryl, isobutyryl, 2-methylburytyl, tigloyl, and downstream species propionyl, and acetyl-carnitine. The method is able to distinguish between isomers of butyryl/isobutyryl carnitines, and valeryl/isovaleryl carnitines. We confirmed the downregulation of BCAA-CARs during BCAA starvation, except for acetyl-carnitine (Fig. 5a), which is not exclusively specific for BCAA catabolism. Interestingly, levels of butyryl and valeryl-CAR, products of β-oxidation, were not changed. Levels of BCAA-CARs were rescued by BCKAs and carnitine treatment (Fig. 5b), but not with acetate, methyl-pyruvate, pantothenic acid, or dimethyl 2-oxoglutarate (metabolites negatively correlated with TG). Similarly, BCKAs rescued C16-BODIPY import into mitochondria with -BCAA treatment, as did free carnitine and BCAA treatment overnight (Fig. 5c), but not pantothenic acid and dimethyl 2-oxoglutarate (Fig. S5). These data confirm that the oxidation of BCAAs in mitochondria results in the formation of the typical SC-CAR species and that their decrease during BCAA starvation can be rescued by BCKAs.

### Acetyl-CoA carboxylase regulates BCAA starvation-induced LD formation

**Fig. 6:**
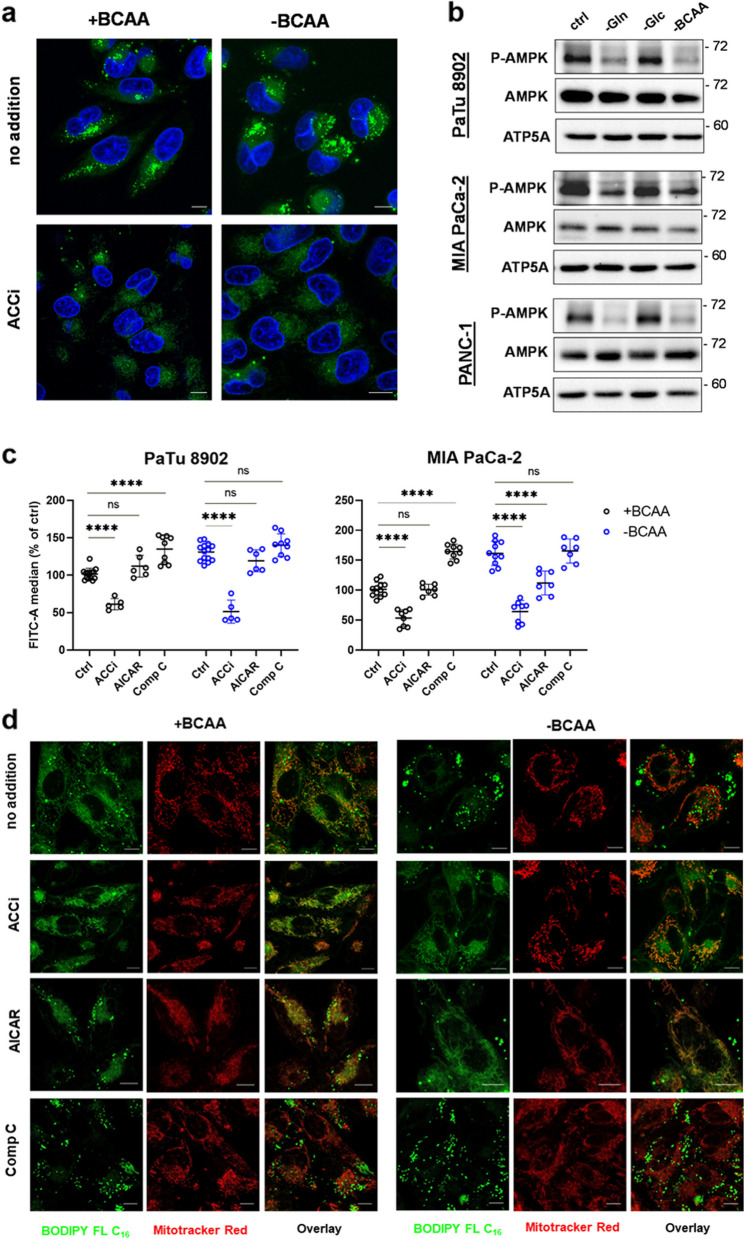
Inhibition of ACC rescues mitochondrial FA import and diminishes LDs. **a** Loss of LDs after ACCi treatment shown by confocal microscopy in MIA PaCa-2 cells. **b** Protein levels of AMPK, and phosphorylated AMPK (P-AMPK, Phospho-AMPKα (Thr172)). **c** Flow cytometry of PaTu 8902 and MIA PaCa-2 cells stained with BODIPY and treated with ACCi (10 µM, o/n), AICAR (500 µM, o/n), and compound C (10 µM, o/n) compared to controls. One-way ANOVA followed by Tukey’s multiple comparisons test. *N* = 3. *n* ≥ 5. **d** C16-BODIPY staining after ACCi, AICAR, and Compound C treatment demonstrated by confocal microscopy in MIA PaCa-2 cells

LD accumulation has been observed previously in response to starvation and the activation of autophagy flux, ER stress, and dysregulation of mTORC1 [25, 27, 31, 32]. Another conserved mechanism of handling metabolic fluxes during a substrate starvation is AMPK signaling [33–35]. One AMPK target is the acetyl-CoA carboxylase (ACC), the product of which is malonyl-CoA, an effective inhibitor of CPT1 [34]. The phosphorylation of ACC by activated AMPK suppresses its activity, which leads to downregulation of malonyl-CoA levels and to release of potential CPT1 inhibition. To test the impact of ACC activity on BCAA starvation-induced LD formation, we assayed BODIPY fluorescence after treatment with ACC inhibitor (TOFA, ACCi). We observed a substantial decrease in BODIPY fluorescence in BCAA-depleted conditions (Fig. 6a, c), confirming that CPT1 inhibition contributes to the formation of LDs in the absence of BCAAs. Next, we have tested AMPK phosphorylation status (i.e. activation status) and observed that AMPK phosphorylation is suppressed in BCAA-starving cells (Figs. 6b, S6b). We have subsequently quantified BODIPY fluorescence using ACCi, AMPK activator AICAR, and AMPK inhibitor Compound C (Comp C). In BCAA-depleted conditions AICAR suppressed LDs to control levels. In contrary, Comp C induced BODIPY fluorescence in control cells but not in BCAA-starving cells (Fig. 6c). Moreover, ACCi and AICAR inhibited LD staining from C16-BODIPY in favor of mitochondrial import, both in BCAA-enriched and also in BCAA-starved cells (Fig. 6d), while Comp C completely halted the import of C16-BODIPY into mitochondria. To verify that this activation/inhibition axis is valid in our cell system, we starved cells of glucose and glutamine and verified C16-BODIPY import (Fig. S6a). As expected, the withdrawal of glucose led to the import of fluorescent dye into mitochondria, while glutamine starvation resulted in LD formation and a minimal import of C16-BODIPY into mitochondria. These data demonstrate that mitochondrial import of FAs is inhibited in BCAA-starving cells due to changes of ACC and AMPK activities.

### TG synthesis protects against detrimental effects of LC-CAR overload in mitochondria

**Fig. 7:**
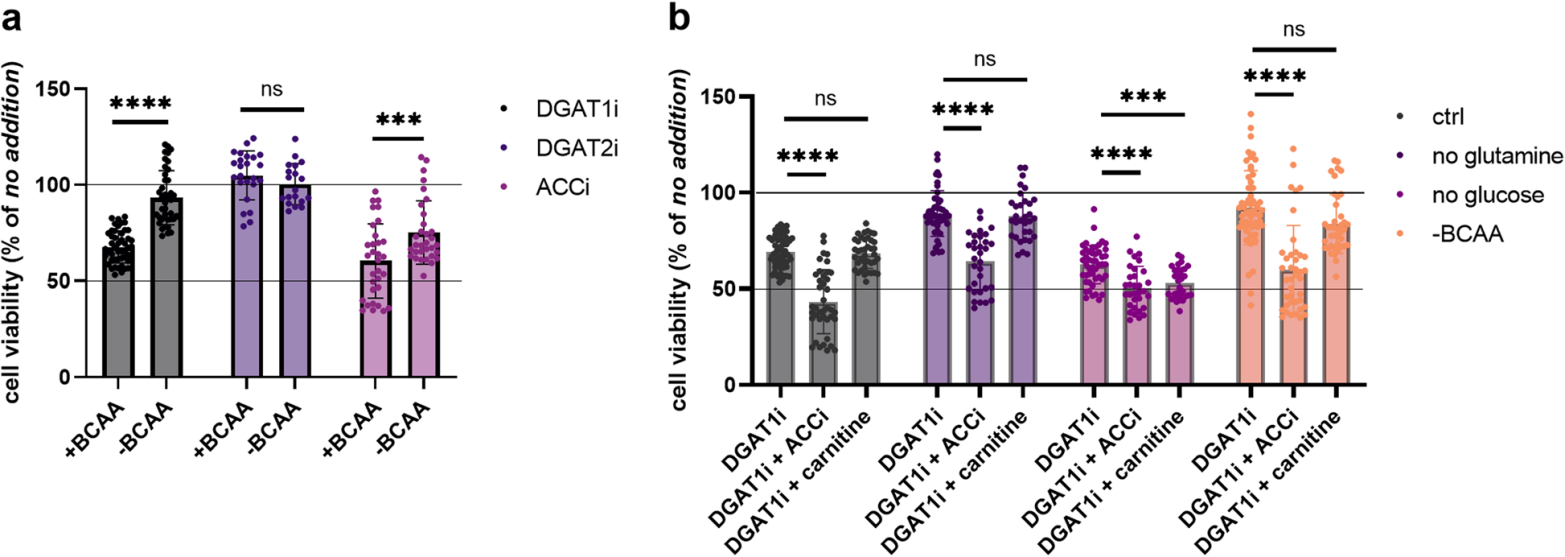
Import of FAs into mitochondria negatively affects cell viability. **a** Cell viability of MIA PaCa-2 cells treated 72 h with DGAT1i, DGAT2i, and ACCi in BCAA-containing or deficient medium. Values are percent of respective control with no addition of pharmacological inhibitor. One-way ANOVA followed by Tukey’s multiple comparisons test. *N* > 3, *n* > 12. Criteria of significance: ***p < 0.001, ****p < 0.0001. **b** Cell viability of MIA PaCa-2 treated with DGAT1i, DGAT1i + ACCi and DGAT1i + carnitine in complete medium, no glutamine, no glucose, and BCAA-depleted cells (-BCAA). Values are percent of respective control with no addition of pharmacological inhibitor. One-way ANOVA followed by Tukey’s multiple comparisons test. *N* > 3, *n* > 20. Criteria of significance: ***p < 0.001, ****p < 0.0001

Further, we asked whether LD formation is part of the adaptive phenotype that helps cells to survive BCAA starvation. It is generally accepted that TG synthesis might neutralize the potentially toxic effects of non-esterified FAs in the cytosol, particularly polyunsaturated FAs (PUFAs), which are prone to oxidation and can cause cellular lipotoxicity [36–38]. Indeed, PUFA levels were elevated to a different extent in BCAA-starved cells, especially in MIA PaCa-2 cells (Fig. S7a). FAs esterified within the TG pool were also considerably less saturated(Fig. S7b). If TG synthesis neutralizes potential lipotoxicity, the inhibition of DGAT1 would be more harmful in cells exhausted of BCAAs. On the other hand, the report of Nguyen et al [25] implied that DGAT1-induced toxicity in starving cells results from the direct import of FAs (LC-CARs) into mitochondria without previous deposition into TGs, causing a ”selective lipotoxic dysfunction of mitochondria”. If applied to our system, DGAT1i treatment would be more detrimental to cells that are able to import FAs into mitochondria, i.e. cells under complete conditions and glucose-starved, but not glutamine-starved or BCAA-starved cells. The inhibition of TG synthesis was significantly more detrimental to cells with BCAAs in the media, in which LD build-up is less apparent (Fig. 7a). It appears that the induced formation of LDs in the absence of BCAAs is a consequence of hindered FA import into mitochondria, but not a response to cytosolic lipotoxicity. Moreover, BCAA-starved cells are less sensitive to ACCi treatment (induction of mitochondrial FA import), perhaps because of lower FA load in the mitochondria prior treatment, as also suggested by Nguyen et al. [25].

The inhibition of LD formation with DGAT1i is supposed to redirect FAs into mitochondria, which causes mitochondrial lipotoxicity, which, in turn, can be prevented in the absence of BCAAs or glutamine. To validate this idea, we examined whether halting the inhibition of CPT1 would sensitize cells to DGAT1 inhibition. ACCi rescued the import of C16-BODIPY into mitochondria in BCAA-starved cells (Fig. 6d). The negative effect of ACCi in combination with DGAT1i was more apparent in glucose-starved cells or cells in complete medium, but the additive effect was clear in all tested cell lines (Fig. 7b). Hence, the inhibited import of LC-CARs into mitochondria in cells that are starved of glutamine or BCAAs has a protective effect compared to cells able to import FAs readily. This line of evidence indicates that inhibition of LD formation and enforcing the import of activated FAs into mitochondria is potentially harmful in terms of cell viability, and that the level of detrimental effect is dependent on the pre-existing mitochondrial load of imported FAs.

In summary, we demonstrated that both mitochondria and LDs serve as a buffering compartment of FAs released during autophagy. As a consequence of inhibited TG synthesis, the free FAs are transported into mitochondria, which causes concomitant cytotoxicity. Lack of certain oxidative substrates, such as valine, leucine, isoleucine, or glutamine, however, is able to inhibit FA import into mitochondria, potentially via the AMPK-ACC-CPT1 axis and protects against the lipotoxic dysfunction of mitochondria.

## Discussion

Systemic BCAA fluctuations accompany PDAC progression, and also the development of cancer cachexia associated with PDAC [39]. The importance of BCAAs for PDAC tumor growth is still unclear; there are studies showing both increased and negligible metabolic utilization of BCAAs by PDAC cells in vitro and in vivo [8–10, 12, 13, 23, 40]. Therefore, we analyzed the interaction of BCAA metabolism with other metabolic pathways and demonstrated that BCAA starvation of PDAC-derived cells results in enhanced TG synthesis and LD biogenesis, probably as a consequence of ongoing autophagy, generating FAs. LD formation in non-adipose tissues represents a strategy to avoid FA excess in the cytosol after various metabolic cues, while lipolysis followed by β-oxidation is a primary mechanism for utilizing FAs released from TGs. Here, we discuss that the downregulated import of LC-CARs into mitochondria in the absence of BCAAs also contributes to LD biogenesis.

To estimate the metabolic function of BCAAs, we assessed bioenergetics and metabolomics features of three cell lines derived from human PDAC with various genetic backgrounds. Surprisingly, we found that BCAA depletion leads to TG synthesis and to a lower extent also CEs, which results in LD accumulation (Figs. 2a, b and 3a, b). This is in contrast to observations reported for the heart muscle, where TG pool was diminished after BCAA-restricted chow diet in Zucker fatty rats [41]. Nevertheless, our model can be considered as an alternative to amino acid starvation at the cellular level, which was shown to trigger the consumption of cellular membranes and subsequent LD biogenesis [25, 26]. Even though we did not inspect details of autophagy initiation under BCAA depletion, our data imply that BCAA starvation itself is capable to trigger autophagy, similarly to HBSS treatment [25, 26]. The induction of autophagy in PDAC cells was also described in response to glutamine or glucose removal [42, 43]. The innovation of our work stems from the experimental evidence demonstrating that DGAT1-dependent TG synthesis in BCAA-depleted cells is a consequence of the impaired delivery of activated FAs into mitochondria, and its redirection into anabolic reactions. Using a series of approaches, we have demonstrated the reciprocity between FA utilization in mitochondria and FA storage in the TG pool, and vice versa (Figs. 4b, c, S4e). Thus, compared to previous reports, our study reveals a novel cellular mechanism leading to LD accumulation, i.e. inhibited import of cytosolic FAs into mitochondria, which leads to LD biogenesis.

To explain this phenomenon, we contemplated three mechanisms that would lead to the uncoupled FA import into mitochondria, i.e. the inhibition of CPT1, inhibition of CACT by the lack of BCAA-derived carnitines, and inhibition of ꞵ-oxidation in mitochondria (Fig. S4b). Several causalities point to the CPT1 inhibition under BCAA deficiency, such as; (i) the lack of mitochondrial targeting of C16-BODIPY, (ii) a decline of LC-CAR instead of the accumulation, and (iii) responsiveness to ACC inhibitor TOFA as the only agent able to rescue mitochondrial targeting of C16-BODIPY. TG synthesis associated with CPT1 inhibition was also demonstrated in several in vitro cell models, including PANC-1 cells [44]. On the other hand, using the model of actinic keratosis and cutaneous squamous cell carcinoma, it has been demonstrated that CACT inhibition results in LC-CAR build-up [30]. Importantly, a negative correlation of LC-CARs and TGs has been noticed by others [45, 46]. This supports our conclusion regarding CPT1 inhibition rather than CACT inhibition in BCAA starvation (see model situations depicted in Fig. S4b**)**. As for the mechanism of CPT1 inactivation, we presume that CPT1 might be inhibited via malonyl-CoA synthesized by ACC, which is a conserved pathway of CPT1 activity regulation [33–35]. For instance, under glucose starvation AMPK phosphorylates and inhibits ACC, which leads to the release of CPT1 inhibition and the metabolic switch to FA oxidation [33]. In our case, glucose removal also readily promoted FA import into mitochondria (Fig. S6a). In contrast, TOFA, an inhibitor of ACC, restored the mitochondrial import of FAs (Fig. 6d**)**, as well as AMPK inhibitor AICAR, which is in line with our hypothesis of CPT1 inhibition via AMPK signaling under BCAA depletion. Apart from TOFA and AICAR, the restoration of mitochondrial import was only achieved with BCKAs and partially also free carnitine. We are, however, aware that the resolution of confocal microscopy does not allow us to definitely distinguish between mitochondrial intermembrane space (IMS) or matrix targeting of C16-BODIPY (Fig. 4b). Despite our argumentations being considered preliminary due to the use of pharmacological inhibitors and in vitro nature of our study, we were able to confirm several steps of the cascade that point to CPT1 inhibition, including the decreased AMPK phosphorylation during BCAA depletion (Fig. 6b). Further genetic manipulations are needed to further elaborate on the exact mechanism of inhibited FA import into mitochondria during BCAA depletion.

Subsequently, we asked what the benefit would be of restraining FA import into mitochondria under BCAA starvation. Our data support the hypothesis that non-lipolysis-derived FA import into mitochondria is potentially detrimental to mitochondrial function [25, 45]. In our case, the inhibition of DGAT1 was more harmful to cells that were able to import FAs into mitochondria, i.e. BCAA-supplemented cells with or without glucose, and it was less harmful to cells that were not able to import FAs into mitochondria, i.e. BCAA- and glutamine-starved cells. We might speculate that LC-CARs imported into mitochondria exceed the capacity of ꞵ-oxidation, so the protective phenotype against DGATi in BCAA-starving cells stems from the low import of LC-CARs in the mitochondria rather than the ability to maintain LDs. Accordingly, the activation of FA import in BCAA-depleted cells with ACCi (TOFA) treatment also decreased cell viability. In light of this, LDs in non-adipose tissues might serve as buffering compartments, which sequester free cytosolic FAs that are not imported into mitochondria. On the other hand, it has been suggested that the pool of FAs released from TGs by lipolysis is directed into mitochondria without any specific toxicity [26]. All these observations suggest that promoting a direct mitochondrial FA uptake after DGAT1 inhibition exceeds the capacity of β-oxidation to process LC-CARs, which negatively affects cell viability. Therefore, LDs can be considered to be a transient cellular compartment that regulates the FA pool in the cytosol and its distribution in the cell and mitochondria.

There is, however, also a controversy regarding the role of BCAA-CARs in our model system. We attempted to demonstrate that catabolic products of BCAAs might represent an actual mechanistic link to FA import into mitochondria and subsequent TG accumulation. The reasoning behind the hypothesis is that BCAA-CARs might be involved in the CACT-mediated exchange of LC-CARs as opposed to free carnitine. In principle, BCAAs are catabolized into SC-CoAs, which are further oxidized or acted on by the enzyme termed CRAT. The primary function of CRAT is to avert R-CoA accumulation by producing BCAA-CAR and SC-CAR intermediates, which can be exported from mitochondria [7, 47]. We are intrigued by the question whether the lack of these BCAA-derived SC-CARs is the primary cause of stalled LC-CAR import into mitochondria and if their dysbalance could initiate a chain of events leading to CPT1 inhibition and TG accumulation. Importantly, there is a report by Long et al., which demonstrated a negative correlation of TG accumulation with BCAA-derived carnitines after iron depletion by deferiprone [48], suggesting that the SC-CAR equilibrium can be disturbed by mechanisms other than BCAA starvation. The link of BCAA-related mitochondrial carnitines and TG synthesis deserves further investigation, ideally in the context of mitochondrial dysfunction, because there is accumulating evidence that enhanced TG synthesis and LD biogenesis correlate with mitochondria-related disturbances [26, 49–51].

Asking whether BCAA species are actually the oxidative substrates in PDAC cells, we favor the interpretation that BCAA metabolism supports ꞵ-oxidation via the import of FAs into mitochondria. We did not detect any solid signal of stable isotope labeled (^13^C) valine and leucine in TCA metabolites (not shown), which is in line with [40]. Besides the decreased respiratory rate and decreased TCA cycle intermediates, we did not observe any consistent changes in mitochondrial markers, suggesting that mitochondrial biogenesis is not affected by BCAA starvation. The oxidation of palmitate was decreased in BCAA-depleted cells (Figs. 4a, S4a); however, our experiments cannot show whether the capacity of ꞵ-oxidation was diminished, or whether its substrate levels are compromised due to decreased import. We are, however, aware of the obvious ambiguity of such an interpretation, but are unable to confirm our hypotheses using available approaches and methods.

The limitation of our study is that we have failed to show the detailed molecular mechanism that would link the FA import to BCAA oxidation products. Also, based on the available data, we cannot conclude whether the proposed mechanism is unique for BCAA starvation, or if it represents a general pathway of substrate redistribution in response to autophagy. Additionally, the role of BCAA-derived carnitines and the role of AMPK-ACC axis in CPT1 inhibition deserve further exploration. Future research using genetic approaches could reveal the respective physiological and pathological applicability. Our study, however, is relevant to substrate variations that have been demonstrated during PDAC progression [39].

## Conclusions

In summary, we have presented the novel aspect of BCAA catabolism in terms of interactions with other mitochondrial and extramitochondrial pathways, both anabolic and catabolic, and provided a novel insight into the mitochondrial-cytosolic crosstalk mediated by metabolic signaling. In our study, BCAA starvation is manifested as LD accumulation, resulting from altered dynamics of FA distribution between oxidation and storage. Mechanistically, the studied phenotype stems from the inhibited import of LC-CARs into mitochondria, most probably due to the diminished AMPK signaling, which leads to ACC activation and the downstream inhibition of CPT1. Activated FAs are subsequently redirected into TG synthesis and sequestered in LDs. We conclude that a decreased consumption of BCAAs can regulate the flux of FAs into β-oxidation and protects against mitochondrial substrate overload.

### Supplementary Information

**Supplementary Material 1.**

**Supplementary Material 2.**

## Data Availability

TIC-normalized metabolomics and lipidomics datasets are provided within Supplementary Material 2. This study includes no data deposited in external repositories. Spectra of the optimized method for BCAA-derived carnitine species detection are available upon request (KG or KS). Other raw data are available upon request (KG or KS).

## Abbreviations

3-MA: 3-methyladenine
ACC: acetyl-CoA carboxylase
AICAR: 5-aminoimidazole-4-carboxamide-1-β-D-ribofuranoside
AMPK: adenosine monophosphate-activated protein kinase
BafA: bafilomycin A1
BCAA: branched-chain amino acid
BCAA-CAR: BCAA-derived carnitine
BCKA: branched-chain keto acid
CACT: carnitine transporter
CE: cholesteryl-ester
Comp C: compound C
CPT1: carnitine palmitoyltransferase 1A
CQ: chloroquine
CRAT: carnitine O-acetyltransferase
DG: diacylglycerol
DGAT: diacylglycerol O-acyltransferase
ETO: etomoxir
LC-CAR: long-chain carnitine
LD: lipid droplet
mTORC1: mammalian target of rapamycin complex 1
PDAC: pancreatic ductal adenocarcinoma
PLS-DA: partial least squares discriminant analysis
SC-CAR: short-chain carnitine
TG: triglyceride
TOFA: 5-(tetradecyloxy)-2-furoic acid
