# Supplementary material for: BCAA metabolism in pancreatic cancer affects lipid balance by regulating fatty acid import into mitochondria
Source: Cancer Metab. 2024 Mar 26;12:10. doi: 10.1186/s40170-024-00335-5 (PMC10967191; doi:10.1186/s40170-024-00335-5)
Supplement: Supplementary file 1 — Supplementary Material 1. [file 40170_2024_335_MOESM1_ESM.pdf]

Supplementary Material 1

BCAA metabolism in pancreatic cancer affects lipid balance by regulating fatty acid import into mitochondria

Klára Gotvaldová[1], Jitka Špačková[1], Jiří Novotný[1], Kamila Baslarová[1,2], Petr Ježek[1], Lenka Rossmeislová[3,4], Jan Gojda[4,5], Katarína Smolková[1,*]

[1]Institute of Physiology of the Czech Academy of Sciences, Laboratory of Mitochondrial Physiology, Prague, Czech Republic

[2]Charles University, First Faculty of Medicine, Prague, Czech Republic

[3]Department of Pathophysiology, Center for Research on Nutrition, Metabolism, and Diabetes, Third Faculty of Medicine, Charles University, Prague, Czech Republic

[4]Franco-Czech Laboratory for Clinical Research on Obesity, Third Faculty of Medicine, Prague, Czech Republic

[4]Department of Internal Medicine, Královské Vinohrady University Hospital and Third Faculty of Medicine, Prague, Czech Republic

[5]Department of Internal Medicine, Královské Vinohrady University Hospital and Third Faculty of Medicine, Prague, Czech Republic

*Katarína Smolková, katarina.smolkova@fgu.cas.cz, Vídeňská 1083, 142 20 Prague 4 - Krč; Phone: +420296442285

Fig. S1

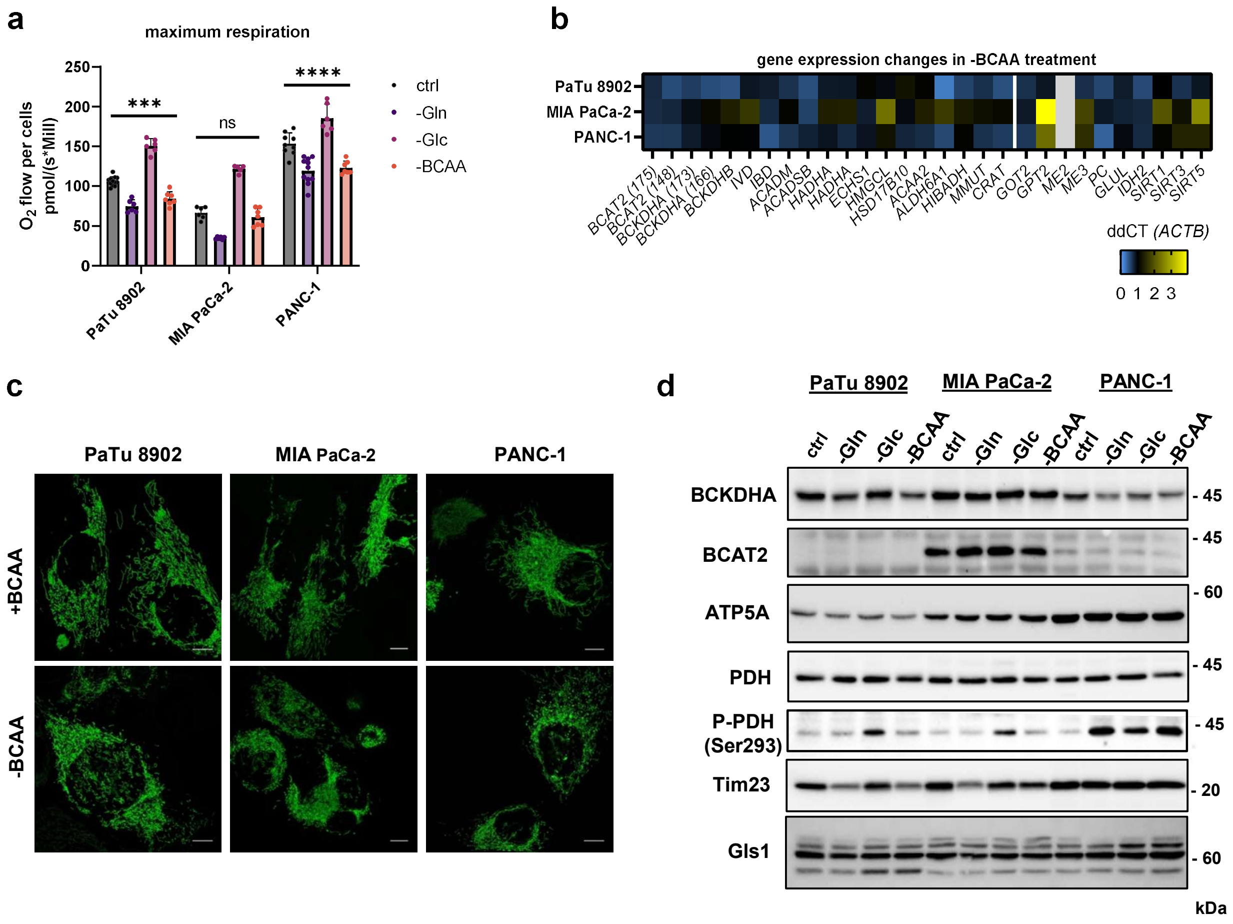

Fig. S1 BCAA starvation affects cell growth and mitochondrial metabolism. **a)** Maximal respiration of intact PDAC cells after addition of carbonyl cyanide-p-trifluoromethoxyphenylhydrazone (FCCP) measured by high-resolution respirometry using the indicated starvation conditions (no glutamine (-Gln), no glucose (-Glc), and and no BCAA (-BCAA)). Two-way ANOVA followed by Tukey's multiple comparisons tests. N>3, n≥6. Criteria of significance: *p<0.05; **p<0.01; ***p<0.001, ****p<0.0001. **b)** Gene expression changes in -BCAA treated cells measured by real-time PCR and expressed as ddCT. List of genes: branched-chain amino acid transaminase 2 (*BCAT2*), branched-chain keto acid dehydrogenase (*BCKDHA*), isovaleryl-CoA dehydrogenase (*IVD*), isobutyryl-CoA dehydrogenase (*IBD*), medium-chain specific acyl-CoA dehydrogenase (*ACADM*), short/branched chain specific acyl-CoA dehydrogenase (*ACADSB*), 2-hydroxyacid dehydrogenase A (*HADHA*), enoyl-CoA hydratase (*ECHS1*), hydroxymethylglutaryl-CoA lyase (*HMGCL*), 3-hydroxyacyl-CoA dehydrogenase type 2 (*HSD17B10*), 3-ketoacyl-CoA thiolase (*ACAA2*), methylmalonate-semialdehyde dehydrogenase 1 (*ALDH6A1*), 3-hydroxyisobutyrate dehydrogenase (*HIBADH*), methylmalonyl-CoA mutase (*MMUT*), carnitine O-acyltransferase (*CRAT*), aspartate aminotransferase 2 (*GOT2*), alanine aminotransferase 2 (*GPT2*), malic enzyme 2 (*ME2*), malic enzyme 3 (*ME3*), pyruvate carboxylase (*PC*), glutamine synthetase (*GLUL*), isocitrate dehydrogenase 2 (*IDH2*), NAD-dependent protein deacetylase sirtuin 1 (*SIRT1*), NAD-dependent protein deacetylase sirtuin 3 (*SIRT3*), NAD-dependent protein deacetylase sirtuin 5 (*SIRT5*). **c)** Confocal microscopy of mitochondrial matrix-targeted GFP transfected into tested cell lines under +BCAA and -BCAA conditions for comparison of potential changes in mitochondrial network. **d)** Western blots of selected mitochondrial markers, BCKDHA, and BCAT2 under the tested conditions, *i.e.* -Gln, -Glc, and -BCAA treatment in comparison to complete medium (*ctrl*). List of proteins: branched-chain keto acid dehydrogenase (BCKDHA), branched-chain amino acid transaminase 2 (BCAT2), ATP synthase F1 subunit alpha, mitochondrial (ATP5A), pyruvate dehydrogenase

(PDH), phosphorylated pyruvate dehydrogenase α1 (P-PDH, Ser293), mitochondrial import inner membrane translocase subunit TIM23 (TIM23), and glutaminase 1 (GLS1)

Fig. S2

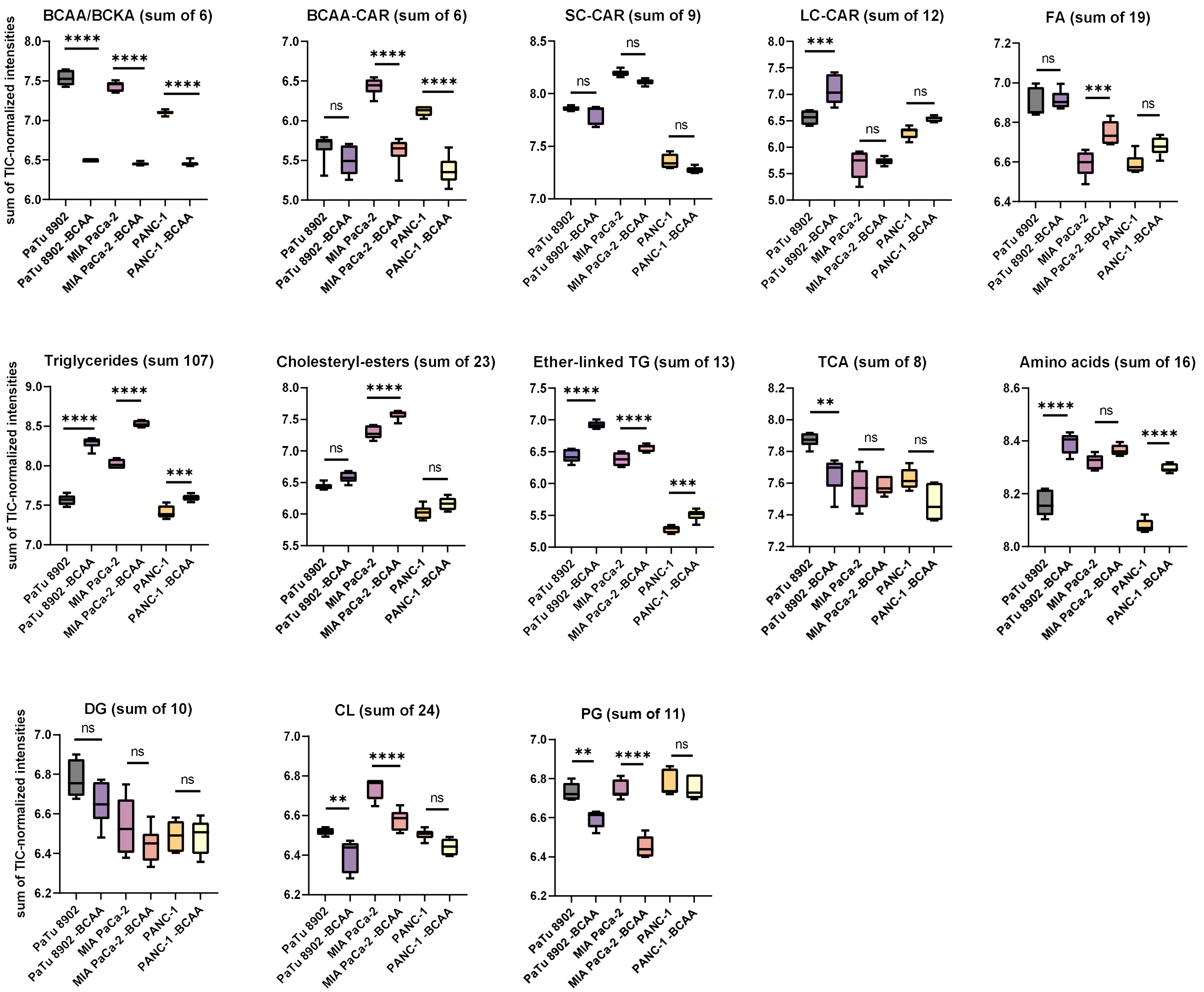

Fig. S2 BCAA starvation induces metabolic remodeling. Box plots of several metabolic classes expressed as the sum of normalized and log10-transformed intensities counting all identified metabolites/lipids, i.e. BCAA/BCKA, BCAA-derived carnitines (BCAA-CAR), short-chain carnitines (SC-CAR), long-chain carnitines (LC-CAR), free fatty acids (FA), triglycerides (TG), cholesteryl-esters (CE), TCA-cycle metabolites (TCA), free amino acids (AA), diacylglycerols (DG), cardiolipins (CL), and phosphoglycerols (PG). One-way analysis of variance (ANOVA) followed by Tukey's multiple comparison tests. N>3, n≥6. Criteria of significance: *p<0.05; **p<0.01; ***p<0.001, ****p<0.0001

Fig. S3

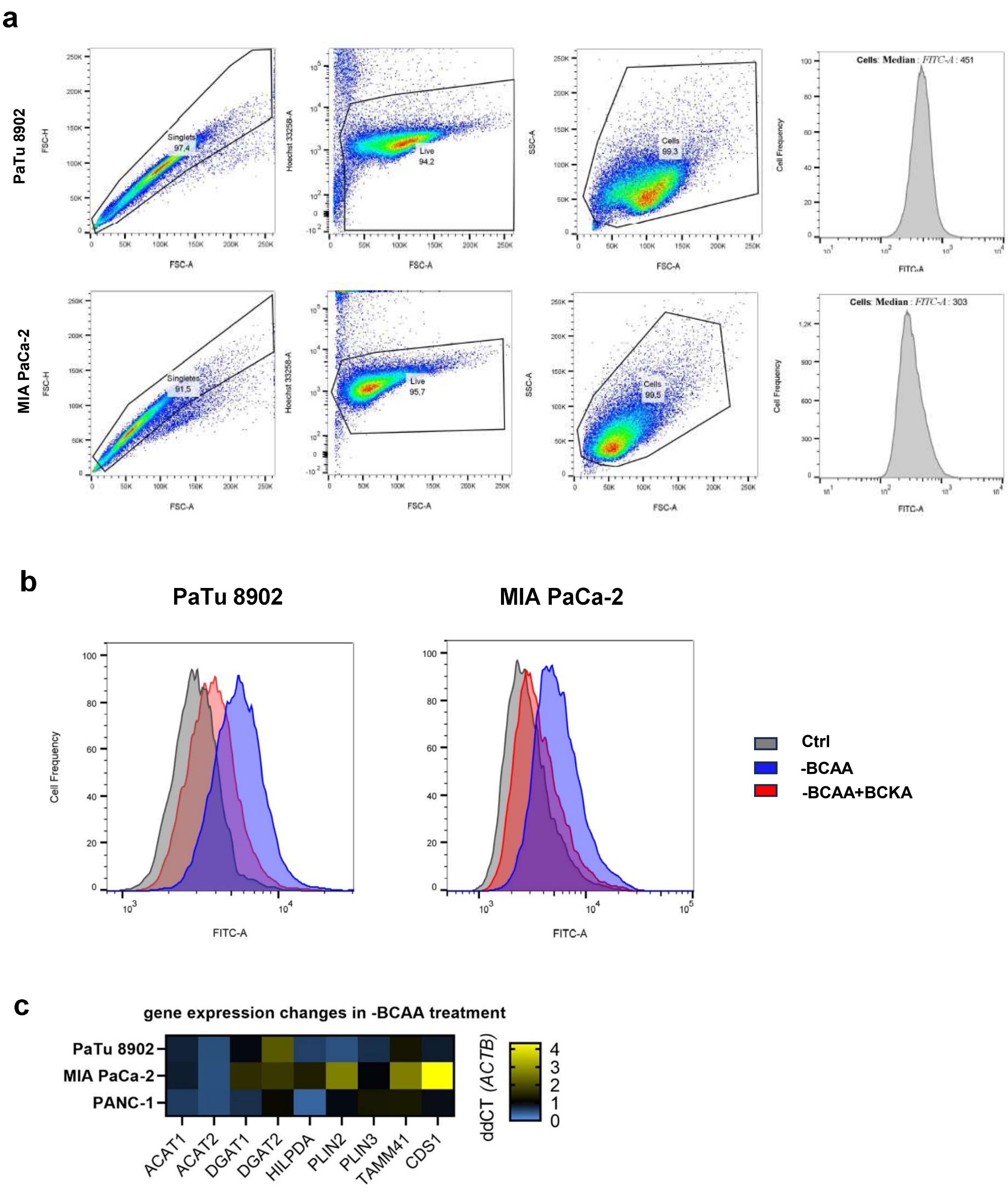

Fig. S3 Triglyceride synthesis and lipid droplets are upregulated in PDAC cells starved of BCAAs. **a)** Gating strategy used for flow cytometry experiments illustrating PaTu 8902 and MIA PaCa-2, histograms depicting mean fluorescence intensity of unstained cells. **b)** Illustrative flow cytometry histograms of PaTu 8902 and MIA PaCa-2; BODIPY™ 493/503 mean fluorescence intensities calculated from 10^4 events. Black for control, blue for -BCAA, and red for -BCAA+BCKA. **c)** Gene expression changes in -BCAA treated cells measured by real-time PCR and expressed as ddCT, namely acetyl-CoA acetyltransferase 1 (*ACAT1*), acetyl-CoA acetyltransferase 2 (*ACAT2*), diacylglycerol O-acyltransferase (*DGAT1*), hypoxia-inducible lipid

droplet-associated protein (*HILPDA*), perilipin-2 (*PLIN2*), perilipin-3 (*PLIN3*), phosphatidate cytidylyltransferase (*TAMM41*), and phosphatidate cytidylyltransferase 1 (*CDS1*)

Fig. S4

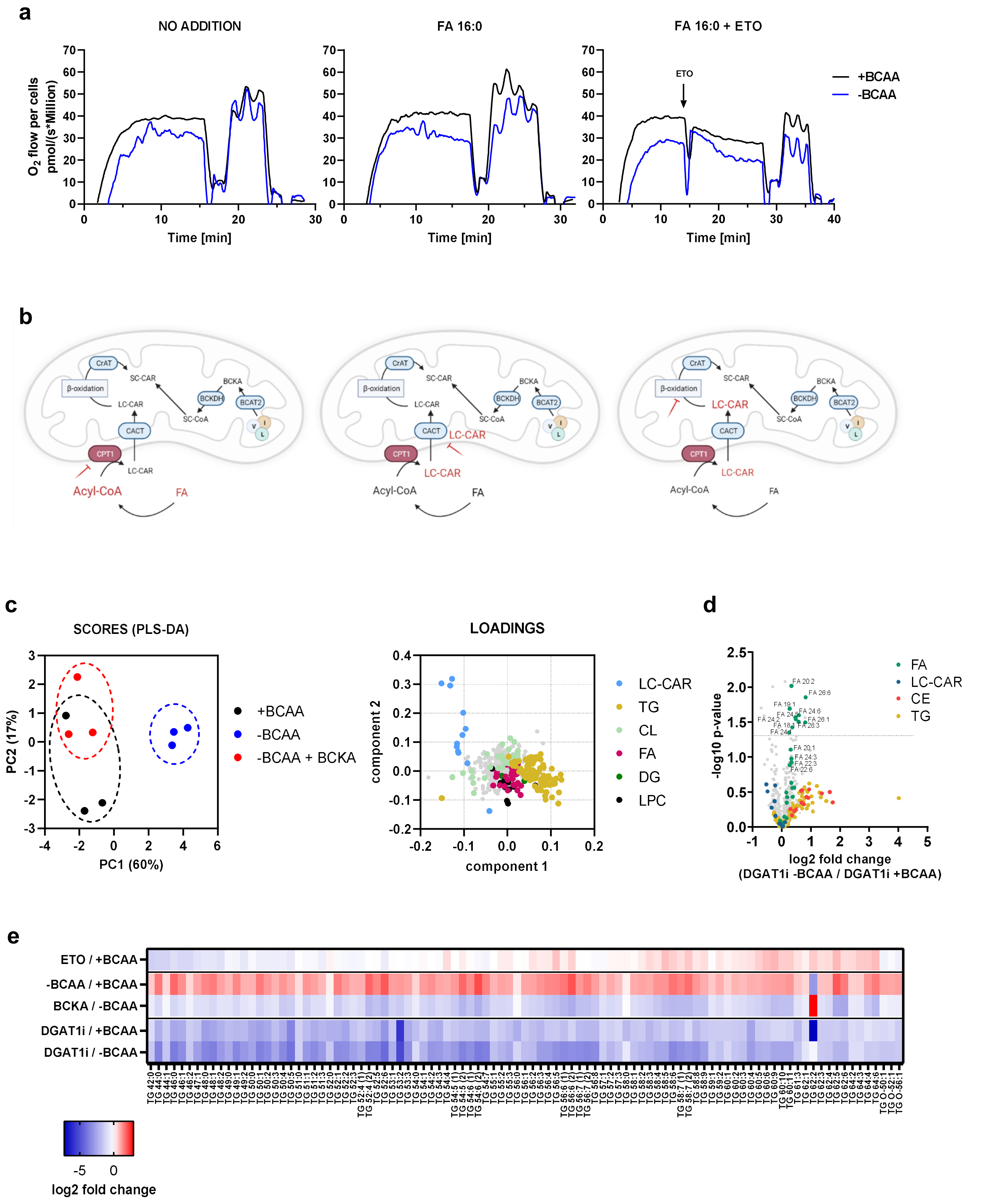

Fig. S4 FA import into mitochondria is diverted into TG synthesis in BCAA-starved cells. **a)** Recorded respiration traces normalized to million cells, performed by high-resolution respirometry. **b)** Schemes depicting scenarios leading to inhibited import of FA into mitochondria in -BCAA treatment, i.e. CPT1 inhibition, CACT inhibition or inhibition (malfunction) of mitochondrial β-oxidation. Created with BioRender.com. **c)** Rescue effect of -BCAA treatment with BCKA in MIA PaCa-2; PLS-DA scores plot

(left) depicting the stratification of samples based on the presence/absence of BCAA in PC3 (t3). The loadings plot corresponding to PLS-DA (right) shows a negative correlation between BCAA/BCKA and TG species. Analyzed with MetaboAnalyst 5.0. **d)** Volcano plot of -BCAA and +BCAA cells treated with DGAT1i shows the build-up of FA in -BCAA cells under tested conditions. **e)** Log2 fold change of TG under designated conditions, namely -BCAA, +BCAA, ETO treatment, and DGAT1i treatment

Fig. S5

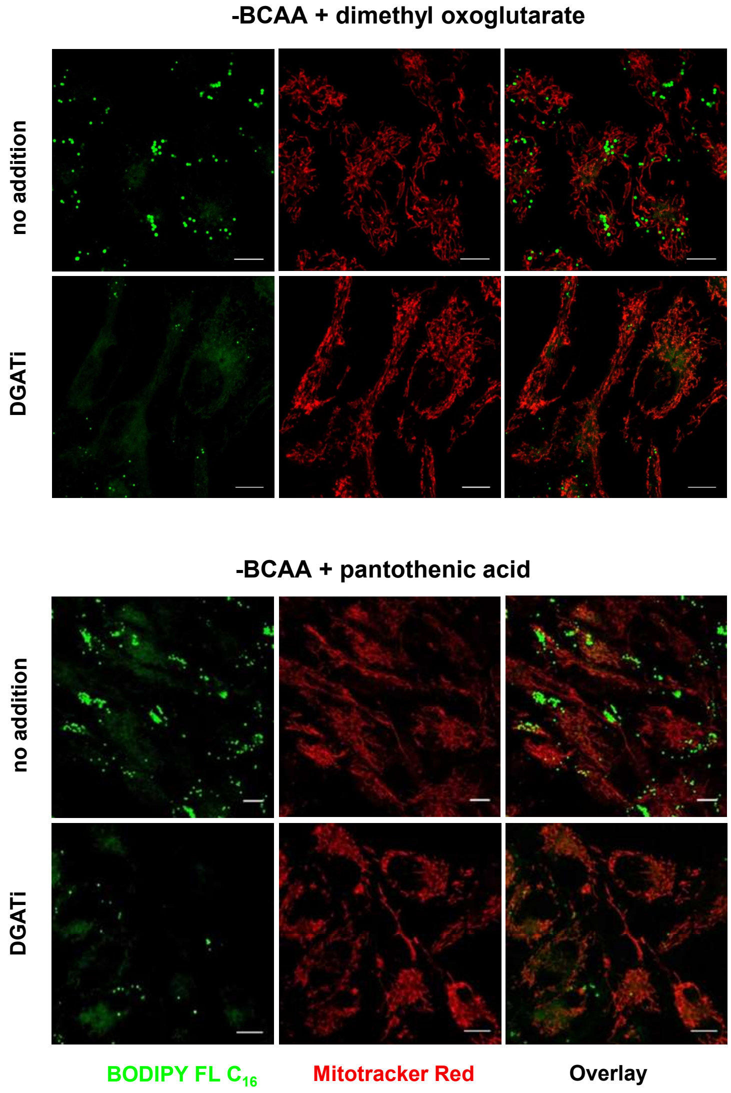

Fig. S5 BCAA-derived carnitines respond as regulators of LC-CAR oxidation. Confocal microscopy demonstrates the lack of rescue effect of import of C16-BODIPY with dimethyloxoglutarate (dmOG) and pantothenic acid (PA), a precursor of coenzyme A

Fig. S6

a

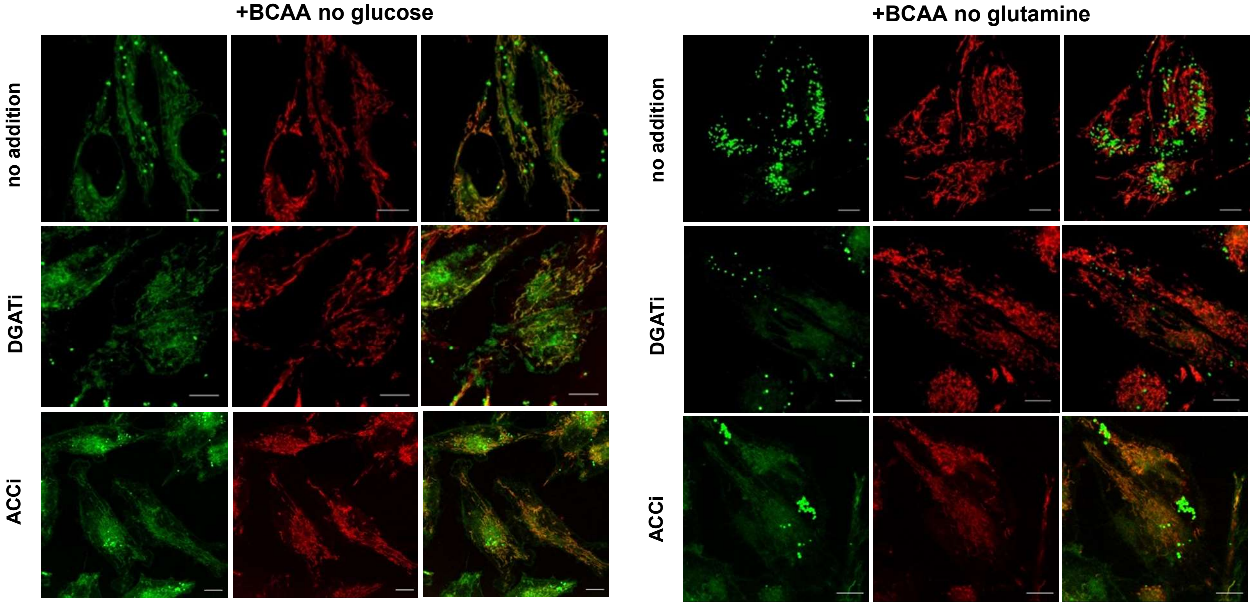

b

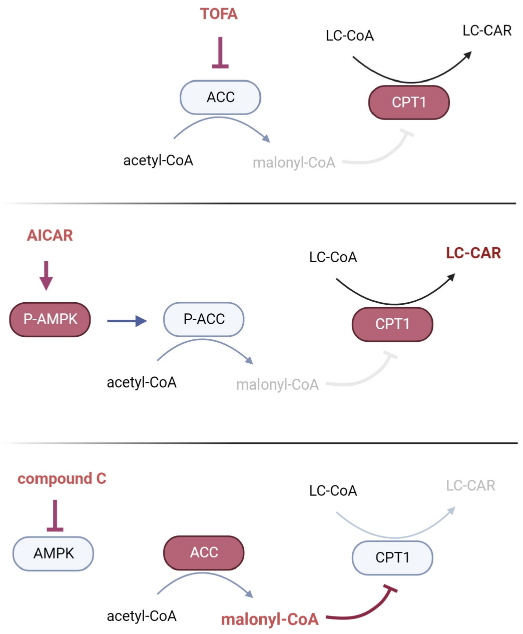

Fig. S6. Import of FAs into mitochondria is regulated by CPT1. **a)** Confocal microscopy images of C16-Bodipy and Mitotracker^{TM} Red with treatment with DGAT1, DGAT2 and ACCi in MIA PaCa-2 cells grown in media without glucose or glutamine. b) Schemes depicting actions of used inhibitors and activators, namely ACCi (TOFA), AICAR (AMPK activator), Compound C (AMPK inhibitor)

Fig. S7

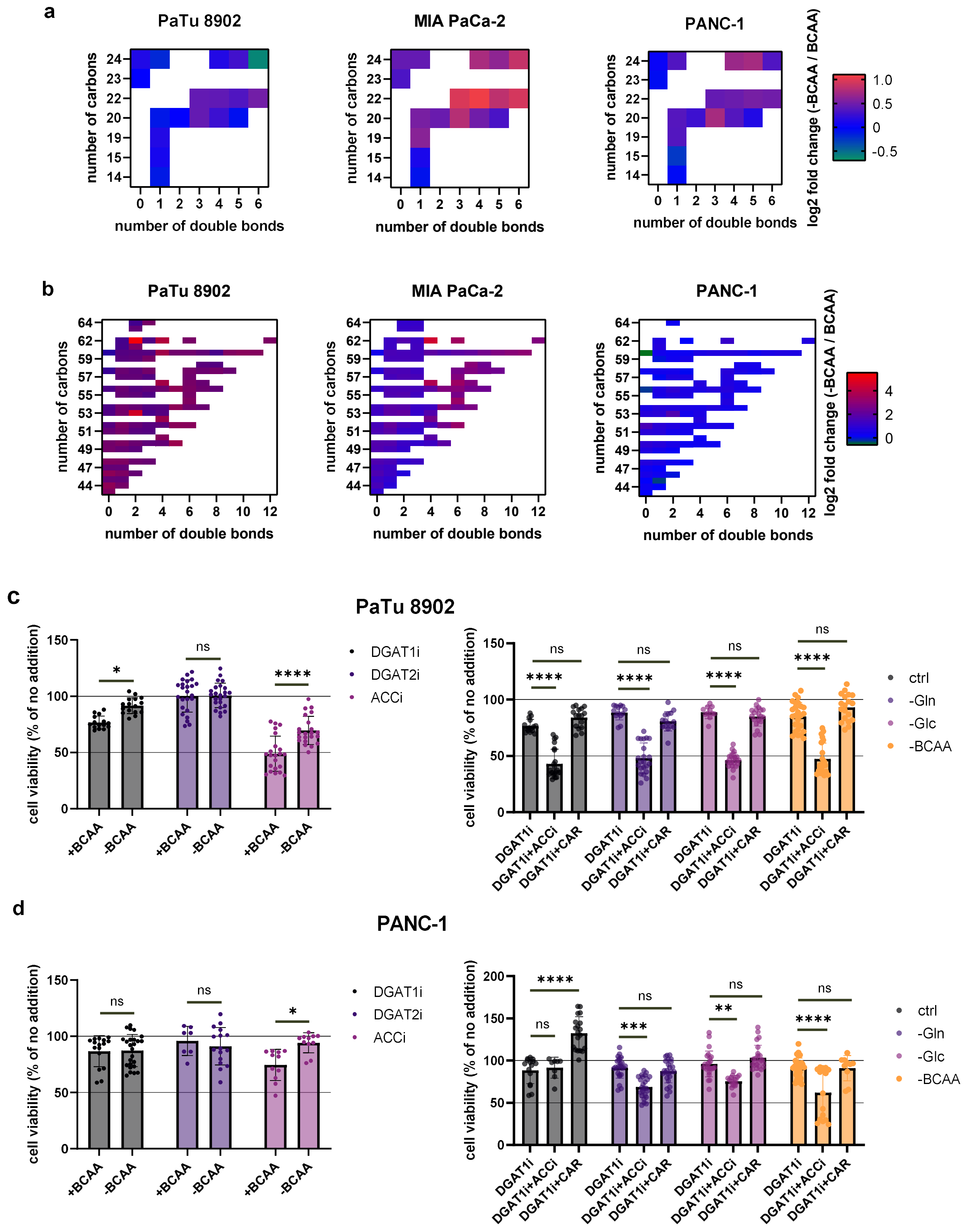

Fig. S7 Import of FA into mitochondria negatively affects cell viability.. **a)** Log2 fold change of the FA pool in -BCAA compared to non-starved cells in PaTu 8902, MIA PaCa-2, and PANC-1 cells; carbon composition and the level of saturation calculated from TIC-normalized data. **b)** Log2 fold change of TG pool in BCAA-free medium compared to non-starved cells in PaTu 8902, MIA PaCa-2, and PANC-

1 cells; carbon composition and the level of saturation calculated from TIC-normalized data. **c)** Left, cell viability of PaTu 8902 cells treated 72 hours with DGAT1i, DGAT2i, and ACCi in BCAA-containing or deficient medium. Right, cell viability of 8902 treated with DGAT1i, DGAT1i + ACCi and DGAT1i + carnitine in complete medium, no glutamine, no glucose, and BCAA-depleted cells (-BCAA). Values are percent of respective control with no addition of pharmacological inhibitor. One-way ANOVA followed by Tukey´s multiple comparisons tests. N>2, n≥8. Criteria of significance: *p<0.05; **p<0.01; ***p<0.001, ****p<0.0001. **d)** Cell viability for PANC-1 cell line treated and evaluated in the same manner as in **Fig. S7c)**

Table S1.

Protein	Gene ID	amplicon length	forward primer 5´ > 3´	reverse primer 5´ > 3´
Branched Chain Amino Acid Transaminase 2	*BCAT2*	175	CACCATGGAAAATGGGCCTG	AGTCATTGGTAGGGAGGCGA
Branched Chain Amino Acid Transaminase 2	*BCAT2*	167	GCGCTCCTGTTCGTCATTCT	TCCTGTTGCACTAACACGGT
Branched Chain Keto Acid Dehydrogenase E1 Subunit Alpha	*BCKDHA*	173	GGAACGCCACTTCGTCACTA	GTGTGGCAGCGAAGTTGAAG
Branched Chain Keto Acid Dehydrogenase E1 Subunit Alpha	*BCKDHA*	257	CTATGAGTCTCAGCGGCAGG	TAGTGACGAAGTGGCGTTCC
Branched Chain Keto Acid Dehydrogenase E1 Subunit Beta	*BCKDHB*	123	GATTTGGAATCGGAATTGCGG	CAGAGCGATAGCGATACTTGG
Isovaleryl-CoA Dehydrogenase	*IVD*	198	TGATGCTGACGTCCTGATTGT	AGGATGTTGGCAGCAGGAATC
Acyl-CoA Dehydrogenase Family Member 8	*ACAD8*	111	GACTTTGCTGCCCGAGAGAT	GTAGACCCCTCCGAAGCCTA
Acyl-CoA Dehydrogenase Medium Chain	*ACADM*	153	GCTACCAAGTATGCCCTGGAA	TCGACGACCAGAATCAACCTC
Acyl-CoA Dehydrogenase Short/Branched Chain	*ACADSB*	262	AAAGGCCACCTATTTGCCTCA	ATGAAGGCCCGGAGTATCAC
Trifunctional enzyme subunit alpha, mitochondrial	*HADHA*	257	AGATGGTGTCCAAGGGCTTC	AAAGACGGCTCCGATGTCTC
Trifunctional enzyme subunit alpha, mitochondrial	*HADHA*	191	AAGTTTTGCTGGGGGCCTTA	TGTCCGTTCCTCTGGAGGTT
Enoyl-CoA Hydratase, Short Chain 1	*ECHS1*	268	TTGATCCAACTGAACCGCCC	CATAGCCATTGACAGCAGCG
3-Hydroxy-3-Methylglutaryl-CoA Lyase	*HMGCL*	260	ACCCCAAATTTGAAAGGCTTCG	CCAAGTCCTGCCACAGAAGA
Hydroxysteroid 17-Beta Dehydrogenase 10	*HSD17B10*	186	GAAAGTTTGGCCGTGTGGAT	CTGGTTCATTCTGGCCCATCT
Acetyl-Coenzyme A acyltransferase 2	*ACAA2*	126	AGACCCCAGCTCTCACGATT	GGCTCATGCTTTCGGTTCCT

Aldehyde Dehydrogenase 6 Family Member A1	*ALDH6A1*	198	GGATTCTGGTGCCCCTGATG	ACTACCCCATGGTTCTTGGC
3-Hydroxyisobutyrate Dehydrogenase	*HIBADH*	243	TGCAGGTGAACAGGTAGTATCTTC	CCAGAAACAGGGGCATCCAT
Methylmalonyl-CoA Mutase	*MMUT*	103	TCCGCCAGTATGCTGGTTTT	TCGCCAGATCAAAGGCAACT
Carnitine O-Acetyltransferase	*CRAT*	120	GCCAAGGCATACAACACCCT	CACGTCTTCTGAGACCCTGG
Glutamic-Oxaloacetic Transaminase 2	*GOT2*	129	CCATGTATTCCAACCCTCCCC	GAGTCCGCATGCCAATGATG
Glutamic-Pyruvic Transaminase 2	*GPT2*	180	CCCATCCCACAATATCCCCTC	GTTCCCAGGGTTGATTATGCAG
Malic Enzyme 2	*ME2*	182	TCAGCCCCAGAGAGCATACC	ATATACACCGACGGTTGGTCT
Carnitine O-Acetyltransferase	*ME3*	114	TGAAGAAGCGCGGATACGATG	GAAAGCAGGGCGGGATTAGG
Pyruvate Carboxylase	*PC*	212	ACAGAGGTGAGATTGCCATCC	CACTGCATCTACGTTGTTCTCC
Glutamate-Ammonia Ligase	*GLUL*	200	TCATCTTGCATCGTGTGTGTG,	CTTCAGACCATTCTCCTCCCG
Isocitrate Dehydrogenase 2	*IDH2*	158	AGTGTACAACTTCCCCGCAG	GCCCATCGTAGGCTTTCAGT
Sirtuin 1	*SIRT1*	224	TGATTGGCACAGATCCTCGAAC	TGAAACAGACACCCCAGCTCC
Sirtuin 3	*SIRT3*	227	ATCGATGGCGAATGAA	ACATGCAGGAGGTATATAAGA
Sirtuin 5	*SIRT5*	137	GAGCTCGCCCACTGTGATTT	TTCGTAGCTGGTGTGGTCTC
Actin Beta	*ACTB*	240	AGAGAGGCATCCTCACCCTG	ATAGCACAGCCTGGATAGCAA
Ribosomal Protein 13	*RLP13*	152	CTGGACCGTCTCAAGGTGTT	TACTTCCAGCCAACCTCGTG
Peptidylprolyl Isomerase A	*PPIA*	155	GTATAAAAGGGGCGGGAGGC	CTGCAAACAGCTCAAAGGAGAC
Glyceraldehyde 3-phosphate Dehydrogenase	*GAPDH*	160	TGAAGACGGGCGGAGAGAAA	GCCCAATACGACCAAATCCGT

Acetyl-CoA Acetyltransferase 1	*ACAT1*	244	AGCATGGGAAGCTGGGAAAT	ATTGAGCCTCTTCGCTGCAT
Acetyl-CoA Acetyltransferase 2	*ACAT2*	204	CCGGAAGATGTGTCTGAGGT	TGCAACCACAATGCTGGAGT
Diacylglycerol O-acyltransferase 1	*DGAT1*	211	CCCCATCCAGGTGGTTTCTC	AACCAGTAAGACCACAGCCG
Diacylglycerol O-acyltransferase 2	*DGAT2*	167	TGTGGCGCTACTTTCGAGAC	GCCTGGGAACTTCTTGCTCA
Hypoxia Inducible Lipid Droplet Associated	*HILPDA*	144	AAGCATGTGTTGAACCTCTACC	TGTGTTGGCTAGTTGGCTTCT
Perilipin 2	*PLIN2*	136	TGCCTGTAAGGGGCTAGACA	CTTGGCCCCAGTCACAGTAG
Perilipin 3	*PLIN3*	167	TTGGGCCAGATGGTGTTGAG	AAGTAGCTCTGTTCCTGCCG
TAM41 Mitochondrial Translocator Assembly and Maintenance Homolog	*TAMM41*	106	ACGACTCCAAAAACCGGTGA	CATGAGGAAAGCAGCGGTCA
CDP-diacylglycerol synthase 1	*CDS1*	106	GTGTTTGGATTCATTGCTGCCT	AGGGCTCACATTCTGTCACG

Table S1. Specification of primers´ sequences used for quantitative PCR as described in Materials and Methods.
